# Association of Prenatal Maternal Anemia With Neurodevelopmental Disorders

**DOI:** 10.1001/jamapsychiatry.2019.2309

**Published:** 2019-09-18

**Authors:** Aline Marileen Wiegersma, Christina Dalman, Brian K. Lee, Håkan Karlsson, Renee M. Gardner

**Affiliations:** 1 Department of Public Health Sciences, Karolinska Institutet, Stockholm, Sweden; 2 Centre for Epidemiology and Community Medicine, Stockholm County Council, Stockholm, Sweden; 3 Department of Epidemiology and Biostatistics, Drexel University Dornsife School of Public Health, Philadelphia, Pennsylvania; 4 A. J. Drexel Autism Institute, Philadelphia, Pennsylvania; 5 Department of Neuroscience, Karolinska Institutet, Stockholm, Sweden

## Abstract

**Importance:**

Given the critical role that iron plays in neurodevelopment, an association between prenatal iron deficiency and later risk of neurodevelopmental disorders, such as autism spectrum disorder (ASD), attention-deficit/hyperactivity disorder (ADHD), and intellectual disability (ID), is plausible.

**Objective:**

To test the a priori hypothesis that anemia diagnosed in mothers during pregnancy is associated with an increased risk of ASD, ADHD, and ID in offspring and that the magnitude of the risk varies with regard to the timing of anemia in pregnancy.

**Design, Setting, and Participants:**

This cohort study used health and population register data from the Stockholm Youth Cohort to evaluate 532 232 nonadoptive children born from January 1, 1987, to December 31, 2010, in Sweden, with follow-up in health registers until December 31, 2016. Data analysis was performed from January 15, 2018, to June 20, 2018.

**Exposures:**

Registered diagnoses of anemia during pregnancy. Gestational timing of the first recorded anemia diagnosis (≤30 weeks or >30 weeks) was considered to assess potential critical windows of development.

**Main Outcomes and Measures:**

Registered diagnoses of ASD, ADHD, or ID or co-occurring combinations of these disorders.

**Results:**

The cohort included 532 232 individuals (272 884 [51.3%] male) between 6 and 29 years of age at the end of follow-up (mean [SD] age, 17.6 [7.1] years) and their 299 768 mothers. The prevalence of ASD, ADHD, and ID was higher among children born to mothers diagnosed with anemia within the first 30 weeks of pregnancy (4.9% ASD, 9.3% ADHD, and 3.1% ID) compared with mothers with anemia diagnosed later in pregnancy (3.8% ASD, 7.2% ADHD, and 1.1% ID) or mothers not diagnosed with anemia (3.5% ASD, 7.1% ADHD, and 1.3% ID). Anemia diagnosed during the first 30 weeks of pregnancy but not later was associated with increased risk of diagnosis of ASD (odds ratio [OR], 1.44; 95% CI, 1.13-1.84), ADHD (OR, 1.37; 95% CI, 1.14-1.64), and ID (OR, 2.20; 95% CI, 1.61-3.01) in offspring in models that included socioeconomic, maternal, and pregnancy-related factors. Early anemia diagnosis was similarly associated with risk of ASD (OR, 2.25; 95% CI, 1.24-4.11) and ID (OR, 2.59; 95% CI, 1.08-6.22) in a matched sibling comparison. Considering mutually exclusive diagnostic groups, we observed the strongest association between anemia and ID without co-occurring ASD (OR, 2.72; 95% CI, 1.84-4.01). Associations of these disorders with anemia diagnosed later in pregnancy were greatly diminished.

**Conclusions and Relevance:**

In contrast to maternal anemia diagnosed toward the end of pregnancy, anemia diagnosed earlier in pregnancy was associated with increased risk of the development of ASD, ADHD, and particularly ID in offspring. Given that iron deficiency and anemia are common among women of childbearing age, our findings emphasize the importance of early screening for iron status and nutritional counseling in antenatal care.

## Key Points

**Question:**

Is maternal anemia during pregnancy associated with risk of 3 commonly co-occurring neurodevelopmental disorders: autism spectrum disorder, attention-deficit/hyperactivity disorder, and intellectual disability?

**Findings:**

In this cohort study of 532 232 nonadoptive Swedish children and their 299 768 mothers, anemia diagnosed earlier in pregnancy (≤30 weeks) was significantly associated with increased offspring risk of autism spectrum disorder, attention-deficit/hyperactivity disorder, and intellectual disability. These associations were not apparent for anemia diagnosed later in pregnancy.

**Meaning:**

The findings suggest that maternal anemia occurring during early pregnancy is associated with increased risk for autism spectrum disorder, attention-deficit/hyperactivity disorder, and in particular, intellectual disability, emphasizing the importance of early screening for iron status and nutritional counseling in antenatal care.

## Teaser

This cohort study examines whether maternal anemia during pregnancy is associated with risk of autism spectrum disorder, attention-deficit/hyperactivity disorder, and intellectual disability in youths.

## Introduction

Iron deficiency and iron deficiency anemia are common during pregnancy, with an estimated prevalence of 30% to 50% for iron deficiency and 15% to 20% for iron deficiency anemia.^1^ Iron demands increase in pregnancy to support the growing fetus and placenta and expand the maternal red blood cell mass.^2^ Severe maternal iron shortage can lead to fetal and neonatal iron deficiency.^3^ Children with neonatal anemia experience cognitive and behavioral deficits, whereas previous animal studies^4^ indicate irreversible neurologic effects of prenatal iron deficiency. Studies of maternal supplemental iron and offspring risk of neurodevelopmental disorders, such as autism spectrum disorder (ASD), have been mixed, with 1 study^5^ indicating a protective association of high intakes of supplemental iron (>86 mg/d) compared with lower levels of iron supplementation (<30 mg/d) and 1 study^6^ reporting no consistent association between iron supplementation and risk of ASD.

Often ASD co-occurs with attention-deficit/hyperactivity disorder (ADHD) and intellectual disability (ID).^7,8^ This comorbid presentation could be the result of shared causes that involve heritable and nonheritable factors occurring during neurodevelopmentally relevant windows.^9,10,11^ The aim of this study was to examine the association between prenatal anemia diagnoses in mothers and offspring risk of ASD, ADHD, and ID. To assess critical windows of development, we considered the timing of anemia diagnosis.

## Methods

### Study Population

**Figure 1.**
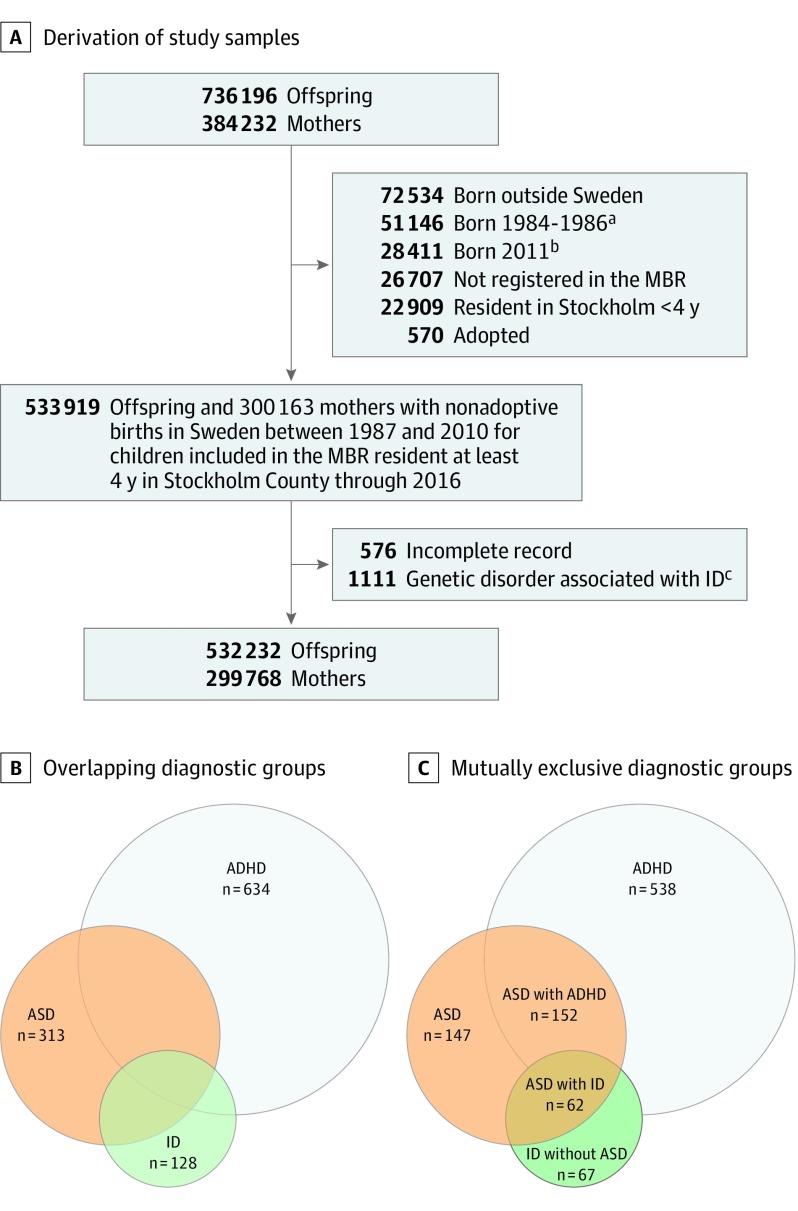
Selection of the Study Population and Characterization of Outcomes in the Stockholm Youth Cohort Less prevalent outcomes (ie, individuals diagnosed with intellectual disability [ID] and attention-deficit/hyperactivity disorder [ADHD] but not autism spectrum disorder [ASD] and individuals diagnosed with all 3 diagnoses) were not considered individually because of insufficient power in these groups. Numbers in panels B and C are cases per 10 000 in the study cohort. ^a^Less than 1% of mothers were diagnosed with anemia from 1984 to 1986, and we excluded people born before 1987 as a result. ^b^Records in the medical birth register (MBR) for individuals born in 2011 were not included in the data linkage for this cohort, and thus we excluded these individuals. ^c^Diagnosed with a known congenital disorder or an inborn error of metabolism that has been associated with intellectual disability.

This cohort study used data from the Stockholm Youth Cohort, a prospective, register-based cohort of individuals born from January 1, 1984, to December 31, 2011, residing in Stockholm County at any point from January 1, 2001, to December 31, 2011.^12^ Data were derived from registers that contain routinely collected health and sociodemographic data cross-linked via each resident’s national identification number. We included all nonadopted individuals born from January 1, 1987, to December 31, 2010, in Sweden, with a complete record in the Medical Birth Register who were residing in Stockholm County for more than 4 years (Figure 1A). We excluded individuals affected by a study outcome who were also affected by a congenital disorder known to be associated with ID (eg, Down syndrome).^13^ Excluded individuals had a lower socioeconomic status compared with included individuals, likely because of the large proportion of migrants in the excluded group (eTable 1 in the Supplement). Data analysis was performed from January 15, 2018, to June 20, 2018. Ethical approval was obtained from the Stockholm Regional Ethical Review Committee, which determined that informed consent was not required for the analysis of anonymized register data.

### Case Ascertainment

*International Classification of Diseases, Ninth Revision*, *International Classification of Diseases, 10th Revision*, and *DSM-IV* codes and information from the Prescription Drug Register (for ADHD medication) were used in previously described^12,14,15,16^ case ascertainment procedures that covered all inpatient and outpatient pathways to care in Stockholm County (eTable 2 in the Supplement), with follow-up until December 31, 2016. We considered 3 potentially overlapping outcomes: any ASD, any ADHD, and any ID (Figure 1B). We also considered 5 mutually exclusive outcomes: ASD only (no ADHD or ID), ADHD only (no ASD or ID), ID without ASD (no ASD, not excluding ADHD), ASD with ID (not excluding ADHD), and ASD with ADHD (no ID) (Figure 1C).

### Anemia

Maternal anemia was defined as an *International Classification of Diseases*–coded diagnosis of anemia complicating pregnancy or iron deficiency anemia (eTable 2 in the Supplement) registered up to 1 calendar year before the birth of the index person, recorded in the Medical Birth Register and the National Patient Register. Anemia diagnosis during the periconceptual period was included because it likely indicates exposure to iron deficiency during early gestation. Hemoglobin level is screened a minimum of 3 times throughout pregnancy (at approximately gestational weeks 10, 25, and 37), with additional measurements if indicated.

To determine critical windows of exposure, the earliest date of anemia diagnosis was considered relative to the gestational day of pregnancy. Gestational week at diagnosis could not be established for 1286 women (4.2% of women who received an anemia diagnosis). Maternal and child characteristics were similar for those missing a diagnosis date compared with those for whom a date was established (eTable 3 in the Supplement).

### Covariates

Covariates were chosen based on prior evidence of an association with the outcomes and were evaluated in our cohort for their association with the exposure (Table 1) and outcomes (eFigure 1 in the Supplement). Disposable income at birth was divided into quintiles, using the complete distribution of disposable income in Sweden and accounting for inflation and family size.^17^ The highest level of education obtained by either parent was categorized as 9 or less, 10 to 12, or more than 12 years of schooling.^17^ Maternal country of origin was dichotomized as born in Sweden or not.

**Table 1.** Selected Characteristics by Maternal Anemia Exposure Category in the Stockholm Youth Cohort (Born 1987-2010)

Characteristic	No. (%) of Participants			
	Maternal Anemia		Diagnosis, wk	
	Yes (n = 31 018)	No (n = 501 214)	≤30 (n = 1534)	>30 (n = 28 198)
Sex				
Male	16 122 (52.0)	256 762 (51.2)	788 (51.4)	14 626 (51.9)
Female	14 896 (48.0)	244 452 (48.8)	746 (48.6)	13 572 (48.1)
Maternal BMI				
Normal (18.5-25)	15 685 (50.6)	252 748 (50.4)	725 (47.3)	14 319 (50.8)
Underweight (<18.5)	800 (2.6)	13 399 (2.7)	52 (3.4)	716 (2.5)
Overweight (25-30)	5579 (18.0)	71 039 (14.2)	250 (16.3)	5101 (18.1)
Obese (>30)	2170 (7.0)	23 675 (4.7)	103 (6.7)	1978 (7.0)
Missing	6784 (21.9)	140 353 (28.0)	404 (26.3)	6084 (21.6)
Maternal age, y				
<25	3933 (12.7)	75 180 (15.0)	251 (16.4)	3503 (12.4)
25-29	8245 (26.6)	148 240 (29.6)	403 (26.3)	7485 (26.5)
30-34	11 188 (36.1)	173 187 (34.6)	501 (32.7)	10 262 (36.4)
35-39	6135 (19.8)	86 675 (17.3)	304 (19.8)	5584 (19.8)
≥40	1517 (4.9)	17 932 (3.6)	75 (4.9)	1364 (4.8)
Disposable income at IP’s birth				
First quintile	4212 (13.6)	71 856 (14.3)	315 (20.5)	3697 (13.1)
Second quintile	6606 (21.3)	105 514 (21.0)	429 (28.0)	5889 (20.9)
Third quintile	6553 (21.1)	109 096 (21.8)	324 (21.1)	5963 (21.2)
Fourth quintile	6734 (21.7)	107 993 (21.6)	247 (16.1)	6228 (22.1)
Fifth quintile	6913 (22.3)	106 755 (21.3)	219 (14.3)	6421 (22.8)
Highest parental education level, y				
≤9	1599 (5.2)	29 014 (5.8)	129 (8.4)	1381 (4.9)
10-12	10 999 (35.5)	191 079 (38.1)	643 (41.9)	9903 (35.1)
>12	17 760 (57.3)	272 670 (54.4)	727 (47.4)	16 325 (57.9)
Missing	660 (2.1)	8451 (1.7)	35 (2.3)	589 (2.1)
Maternal psychiatric history before IP’s birth (any diagnosis)				
Not present	20 544 (66.2)	355 244 (67.8)	908 (59.2)	18 817 (66.7)
Present	10 474 (33.8)	161 733 (32.3)	626 (40.8)	9381 (33.3)
Single or multiple birth				
Single	28 699 (92.5)	488 961 (97.6)	1 360 (88.7)	26 237 (93.0)
Multiple	2 319 (7.45)	12 253 (2.4)	174 (11.3)	1961 (7.0)
Birth order (parity)				
First child	17 320 (55.8)	224 443 (44.8)	575 (37.5)	15 982 (56.7)
Second child	8 934 (28.8)	183 821 (36.7)	540 (35.2)	8074 (28.6)
Third or later child	4 764 (15.4)	92 950 (18.5)	419 (27.3)	4142 (14.7)
Mother hospitalized for infection during pregnancy				
No	28 619 (92.3)	483 099 (96.6)	1 336 (87.1)	26 139 (92.7)
Yes	2373 (7.7)	17 229 (3.4)	198 (12.9)	2059 (7.3)
Mother born outside Sweden				
No	22 141 (71.34)	377 498 (75.3)	862 (56.2)	20 397 (72.3)
Yes	8877 (28.6)	123 716 (24.7)	672 (43.8)	7801 (27.7)
Interpregnancy interval, y				
First born	17 320 (55.8)	224 443 (44.8)	575 (37.5)	15 982 (56.7)
<1	1941 (6.23)	42 557(8.5)	157 (10.2)	1709 (6.1)
1-2	3579 (11.5)	81 891 (16.3)	194 (12.6)	3246 (11.5)
2-5	4500 (14.5)	91 657 (18.3)	307 (20.0)	4037 (14.3)
5-10	1871 (6.0)	33 499 (6.7)	141 (9.2)	1652 (5.9)
>10	539 (1.7)	8465 (1.7)	40 (2.6)	472 (1.7)
Missing	1268 (4.1)	18 702 (3.7)	120 (7.8)	1100 (3.9)
Size for gestational age				
Small for gestational age	684 (2.2)	11 761 (2.4)	92 (6.0)	556 (2.0)
Normal	26 320 (84.8)	460 425 (91.9)	1212 (79.0)	24 121 (85.5)
Large for gestational age	1 547 (5.0)	14 317 (2.9)	47 (3.1)	1447 (5.1)
Missing size for gestational age	148 (0.5)	2458 (0.5)	9 (0.6)	113 (0.4)
Missing because of multiple birth	2319 (7.5)	12 253 (2.4)	174 (11.3)	1961 (7.0)
Low Apgar score (<7)				
No	30 228 (98.2)	492 997 (99.1)	1453 (95.7)	27 543 (98.4)
Yes	547 (1.8)	4363 (0.9)	65 (4.3)	442 (1.6)
Missing	243 (1.8)	3854 (0.8)	16 (1.0)	213 (0.8)
Cesarean delivery				
No	20 585 (66.4)	422 980 (84.4)	956 (62.3)	18 782 (66.6)
Yes	10 433 (33.6)	78 225 (15.6)	578 (37.7)	9416 (33.4)
Missing	0	9 (<0.1)	0	0
Gestational age at birth				
Preterm (induced)	1879 (6.1)	11 948 (2.4)	355 (23.1)	1397 (5.0)
Preterm (spontaneous)	852 (2.8)	14 898 (3.0)	140 (9.1)	678 (2.4)
Term	25 186 (81.3)	437 864 (87.5)	973 (63.4)	23 281 (82.6)
Post term	3075 (9.9)	35 618 (7.1)	66 (4.3)	2842 (10.1)
Missing	26 (0.1)	886 (0.2)	0	0
Preeclampsia				
Yes	2 541 (8.2)	15 459 (3.1)	109 (7.1)	2284 (8.1)
No	28 477 (91.8)	485 755 (96.9)	1425 (92.9)	25 914 (91.9)

Abbreviations: BMI, body mass index (calculated as weight in kilograms divided by height in meters squared); IP, index person.

Maternal body mass index (BMI) was calculated as weight in kilograms divided by height in meters squared from height and weight recorded by midwives at the first antenatal visit.^18^ The interpregnancy interval (IPI; ie, the time between the birth of a child and the conception of the next child) was categorized as less than 1, 1 to 2, 2 to 5, 5 to 10, or more than 10 years and being first born (because first-born children by definition have no IPI). Births were categorized as multiple or singleton. Maternal hospitalization for infection during pregnancy (yes or no)^19^ and maternal inpatient or outpatient psychiatric history before birth of the child (eTable 2 in the Supplement) were extracted from the National Patient Register and regional psychiatry registers.

### Statistical Analysis

Statistical analyses were performed using Stata/SE, version 13.1 (StataCorp). Odds ratios (ORs) were calculated with generalized estimating equation models with logit link clustered on maternal identification number to account for the clustering of siblings born to the same mother in the data set.

To examine the association between maternal anemia and offspring risk for ASD, ADHD, and ID, model 1 adjusted only for sex and birth year. Model 2 accounted for sex; birth year; parental educational level and disposable income; maternal country of origin, BMI, age, psychiatric history, and infection during pregnancy; multiple birth; and IPI, as specified above. This analysis was repeated after categorizing the exposure by time of maternal diagnosis of anemia: earlier diagnosis of anemia (≤30 weeks), later diagnosis of anemia (>30 weeks), or no anemia (referent). We selected the earliest cutoff that included adequate cases of ASD, ADHD, and ID to allow for adjusted analyses. To further examine the role of timing of anemia onset, we used restricted cubic spline models with 3 knots to flexibly fit associations between gestational week at anemia diagnosis and odds of each outcome among the 29 732 women with a dated anemia diagnosis, using week 40 as the referent.

#### Sensitivity Analysis

The prevalence of maternal anemia was substantially lower before 1997 (eFigure 2 in the Supplement), indicating potential underascertainment before 1997.^20^ We repeated our main statistical analyses after stratification on birth year (<1997 or ≥1997).

#### Sibling Analysis

Associations between maternal anemia and offspring risk of neurodevelopmental disorders may be confounded by unobserved factors, such as shared genetic liability. To evaluate the possibility of such unobserved confounding, we used conditional logistic regression models to compare full siblings exposed to anemia (any, ≤30 weeks, or >30 weeks) with nonexposed siblings in terms of risk of any ASD, any ADHD, and any ID diagnoses, adjusted for factors that are often not shared by siblings: sex, birth year, and IPI.

#### Mediation Analysis

Maternal anemia can lead to adverse obstetric outcomes,^21^ which in turn may be associated with increased risk of ASD, ADHD, and ID. We examined mediation by the following covariates: size for gestational age (small for gestational age or *z* score ≤−2, large for gestational age or *z* score ≥2, or normal), low Apgar score (<7) 5 minutes after birth (yes or no), cesarean delivery (yes or no), and gestational age at birth (<37 weeks, 37-42 weeks, or >42 weeks). Preterm birth at less than 37 weeks was further categorized to indicate whether labor started spontaneously or was medically indicated (ie, the result of induction or cesarean delivery). We performed a mediation analysis using the counterfactual framework method to explore mechanisms by which anemia may be associated with increased risk of ASD, ADHD, and ID, adjusted as in model 2, to estimate direct and indirect relationships (via the mediator) in the Stata module PARAMED.^22,23^ The proportion mediated was calculated as log(natural indirect relationship)/log(total relationship).

Because we suspected underascertainment of anemia before 1997 and because nondifferential measurement error of the exposure can bias estimates,^23^ we repeated the mediation analysis excluding individuals born before 1997. Since a complex association between multiple births and adverse birth outcomes exists, multiple births were excluded from the mediation analysis.

## Results

### Study Sample

A total of 532 232 children (272 884 [51.3%] male) between 6 and 29 years of age at the end of follow-up (mean [SD] age, 17.6 [7.1] years) and their 299 768 mothers were included in our study. During 31 018 pregnancies (5.8%), mothers were diagnosed with anemia. Of these diagnoses, 1534 (5.0%) occurred before 30 weeks of pregnancy and 28 198 (90.9%) occurred after 30 weeks of pregnancy (eFigure 3 in the Supplement).

Anemia diagnosis was more common among overweight (OR, 1.16; 95% CI, 1.12-1.19) and obese (OR, 1.28; 95% CI, 1.22-1.34) mothers compared with normal-weight mothers, among mothers older than 40 years compared with mothers younger than 25 years (OR, 1.17; 95% CI, 1.10-1.25), among mothers with a psychiatric history (OR, 1.12; 95% CI, 1.09-1.15), in families in the highest income quintile compared with the lowest (OR, 1.04; 95% CI, 1.00-1.08), in primiparous women (OR, 1.57; 95% CI, 1.52-1.63), and in mothers with an IPI longer than 5 years (OR, 1.15; 95% CI, 1.09-1.21) compared with multiparous women with 2 to 5 years between pregnancies, in multiple births (OR, 3.09; 95% CI, 2.94-3.24), and among mothers who were hospitalized for infection during pregnancy (OR, 2.04; 95% CI, 1.95-2.14) (eTable 4 in the Supplement). In contrast, earlier-onset anemia occurred more often in less educated parents (OR, 0.49; 95%, CI 0.40-0.60, comparing parents with >12 years of education with those with <9 years), in less wealthy families (OR, 0.46; 95% CI, 0.39-0.55, comparing those in the highest income quintile with those in the lowest), among underweight mothers compared with normal-weight mothers (OR, 1.46; 95% CI, 1.09-1.96), and among younger mothers (OR, 0.75; 95% CI, 0.64-0.88, comparing mothers 30-34 years old with those <25 years old).

Children born to mothers with anemia diagnosed at 30 weeks or less were more likely to be born preterm (OR, 7.10; 95% CI, 6.28-8.03) or small for gestational age (OR, 2.81; 95% CI, 2.26-3.50) compared with children whose mothers were not diagnosed with anemia, whereas children whose mothers were diagnosed with anemia at greater than 30 weeks’ gestation were more likely to be born post term (OR, 1.56; 95% CI, 1.49-1.62) and large for gestational age (OR, 1.76; 95% CI, 1.66-1.87) (eFigure 4 in the Supplement).

### Primary Analyses

We observed a small increase in risk of ASD (OR, 1.11; 95% CI, 1.04-1.18), ADHD (OR, 1.06; 95% CI, 1.01-1.11), and ID (OR, 1.12; 95% CI, 1.00-1.24) in offspring of mothers diagnosed with anemia in crude models (Table 2). Risks were also elevated in crude models when considering the mutually exclusive diagnostic groups, although the association was not statistically significant for the diagnoses of ID without ASD, ASD with ID, and ASD with ADHD (Table 2).

**Table 2.** Prevalence of and ORs for ASD, ADHD, and ID in Offspring of Mothers Diagnosed With Anemia During Pregnancy, With Consideration of the Timing of Diagnosis

Outcome	Cases, No. (%) [95% CI]^a^		OR (95% CI)		No .of Siblings (Discordant Pairs, %)^d^	Model 3, OR (95%CI)^e^
	No Maternal Anemia	Maternal Anemia	Model 1^b^	Model 2^c^		
**Anemia (All Prenatal Diagnoses)**						
Any ASD^f^	16 523 (3.30) [3.25-3.35]	1147 (3.70) [3.49-3.91]	1.11 (1.04-1.18)	1.05 (0.99-1.12)	21 949 (4.92/4.34)	1.00 (0.87-1.14)
Any ADHD^f^	34 890 (6.96) [6.89-7.03]	2252 (7.26) [6.97-7.55]	1.06 (1.01-1.11)	1.04 (0.99-1.09)	44 563 (4.64/4.18)	1.01 (0.91-1.11)
Any ID^f^	5904 (1.18) [1.15-1.21]	361 (1.16) [1.05-1.29]	1.12 (1.00-1.24)	1.05 (0.94-1.17)	8 512 (4.18/3.59)	1.10 (0.87-1.38)
ASD^g^	6732 (1.34) [1.31-1.38]	481 (1.56) [1.42-1.69]	1.14 (1.04-1.25)	1.08 (0.98-1.19)	NA	NA
ADHD^g^	26 958 (5.38) [5.32-5.44]	1681 (5.42) [5.17-5.68]	1.05 (1.00-1.11)	1.04 (0.99-1.10)	NA	NA
ID without ASD^g^	3083 (0.62) [0.59-0.64]	180 (0.58) [0.50-0.67]	1.14 (0.98-1.32)	1.06 (0.91-1.24)	NA	NA
ASD with ID^g^	2821 (0.56) [0.54-0.58]	181 (0.58) [0.50-0.67]	1.10 (0.95-1.28)	1.03 (0.88-1.20)	NA	NA
ASD with ADHD^g^	6970 (1.39) [1.36-1.42]	485 (1.56) [1.43-1.71]	1.08 (0.99-1.19)	1.04 (0.94-1.14)	NA	NA
**Anemia Diagnosis ≤30 wk**^h^						
Any ASD^f^	16 523 (3.30) [3.25-3.35]	69 (4.49) [3.52-5.66]	1.54 (1.21-1.96)	1.44 (1.13-1.84)	21 835 (0.29/0.06)	2.25 (1.24-4.11)
Any ADHD^f^	34 890 (6.96) [6.89-7.03]	138 (9.00) [7.61–10.54]	1.48 (1.24-1.77)	1.37 (1.14-1.64)	44 352 (0.20/0.19)	1.18 (0.79-1.76)
Any ID^f^	5904 (1.18) [1.15-1.21]	43 (2.80) [2.04-3.76]	2.85 (2.09-3.89)	2.20 (1.61-3.01)	8 479 (0.22/0.13)	2.59 (1.08-6.22)
ASD^g^	6732 (1.34) [1.31-1.38]	26 (1.69) [1.11-2.47]	1.38 (0.94-2.03)	1.35 (0.92-2.00)	NA	NA
ADHD^g^	26 958 (5.38) [5.32-5.44]	91 (5.93) [4.80-7.23]	1.34 (1.08-1.66)	1.23 (0.99-1.53)	NA	NA
ID without ASD^g^	3083 (0.62) [0.59-0.64]	27 (1.76) [1.16-2.55]	3.57 (2.41-5.27)	2.72 (1.84-4.01)	NA	NA
ASD with ID^g^	2821 (0.56) [0.54-0.58]	16 (1.04) [0.60-1.69]	2.23 (1.36-3.64)	1.74 (1.06-2.86)	NA	NA
ASD with ADHD^g^	6970 (1.39) [1.36-1.42]	27 (1.76) [1.16-2.55]	1.44 (0.99-2.11)	1.38 (0.94-2.03)	NA	NA
**Anemia Diagnosis >30 wk**^h^						
Any ASD^f^	16 523 (3.30) [3.25-3.35]	1014 (3.60) [3.38-3.82]	1.07 (1.00-1.14)	1.02 (0.95-1.09)	21 835 (4.44/4.15)	0.93 (0.81-1.08)
Any ADHD^f^	34 890 (6.96) [6.89-7.03]	1997 (7.08) [6.79-7.39]	1.03 (0.98-1.08)	1.01 (0.96-1.06)	44 352 (4.19/3.83)	0.97 (0.88-1.08)
Any ID^f^	5904 (1.18) [1.15-1.21]	296 (1.05) [0.93-1.18]	1.01 (0.90-1.14)	0.96 (0.85-1.09)	8 479 (4.44/4.15)	1.01 (0.79-1.29)
ASD^g^	6732 (1.34) [1.31-1.38]	435 (1.54) [1.40-1.69]	1.13 (1.02-1.25)	1.07 (0.97-1.18)	NA	NA
ADHD^g^	26 958 (5.38) [5.32-5.44]	1478 (5.24) [4.98-5.51]	1.04 (0.98-1.09)	1.03 (0.97-1.09)	NA	NA
ID without ASD^g^	3083 (0.62) [0.59-0.64]	142 (0.50) [0.42-0.59]	0.99 (0.84-1.18)	0.95 (0.80-1.12)	NA	NA
ASD with ID^g^	2821 (0.56) [0.54-0.58]	154 (0.55) [0.46-0.64]	1.03 (0.87-1.21)	0.98 (0.83-1.15)	NA	NA
ASD with ADHD^g^	6970 (1.39) [1.36-1.42]	425 (1.51) [1.37-1.66]	1.04 (0.94-1.15)	0.99 (0.90-1.10)	NA	NA

Abbreviations: ADHD, attention-deficit/hyperactivity disorder; ASD, autism spectrum disorder; GEE, generalized estimating equation; ID, intellectual disability; NA, not applicable; OR, odds ratio.

^a^ The frequency of each diagnostic outcome, followed by the prevalence (proportion) and 95% confidence interval for the prevalence.

^b^ Model 1: GEE model, clustered on maternal identifier, adjusted only for birth year and sex.

^c^ Model 2: GEE model, clustered on maternal identifier, adjusted for: birth year, sex, educational level, disposable income, mother born outside Sweden, body mass index, maternal age, maternal psychiatric history, multiple birth, interpregnancy interval, and maternal infection during pregnancy.

^d^ Proportion of discordant sibling pairs: the proportion of sibling pairs with the affected sibling exposed and the unaffected sibling unexposed is reported followed by the proportion of sibling pairs with the unaffected sibling exposed and the affected sibling unexposed.

^e^ Model 3: conditional logistic regression model.

^f^ Potentially overlapping diagnostic groups. Individuals included in one diagnostic group are potentially included in another diagnostic group (Figure 1B).

^g^ Mutually exclusive diagnostic groups. Individuals are included in only a single diagnostic group (Figure 1C).

^h^ Pregnancies affected by anemia but with unknown gestational week of diagnosis were excluded from this analysis.

**Figure 2.**
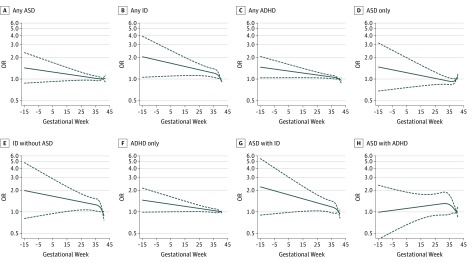
Association Between Gestational Week of Maternal Anemia Diagnosis and Offspring Odds of Neurodevelopmental Outcomes Among the 29 732 Women With a Dated Anemia Diagnosis The odds of each outcome according to gestational week at anemia diagnosis were flexibly fit using a restricted cubic spline model with 3 knots and gestational week 40 set as the referent. The solid line represents the odds ratio (OR) estimated from the fully adjusted generalized estimating equation model, clustered on maternal identifier, and adjusted for birth year, sex, educational level, disposable income, mother born outside Sweden, body mass index, maternal age, maternal psychiatric history, multiple birth, interpregnancy interval, and maternal infection during pregnancy. The dotted lines represent the 95% CI for the fully adjusted model. Results are shown for the potentially overlapping diagnostic outcomes (Figure 1B) in panels A to C and for the mutually exclusive diagnostic categories (Figure 1C) in panels D to H.

After stratification of the exposure by the timing of anemia diagnosis, risk of ADHD (OR, 1.48; 95% CI, 1.24-1.77) and ID (OR, 2.85; 95% CI, 2.09-3.89) was increased among offspring of mothers with an early (≤30 weeks) diagnosis of anemia in crude models (Table 2). In contrast, risk of ADHD (OR, 1.03; 95% CI, 0.98-1.08) and ID (OR, 1.01; 95% CI, 0.90-1.14) were not increased among offspring of mothers with a later (>30 weeks) diagnosis of anemia (Table 2). Risk of offspring ASD was associated with both earlier (OR, 1.54; 95% CI, 1.21-1.96) and later (OR, 1.07; 95% CI, 1.00-1.14) anemia diagnoses, although the association with earlier anemia was more pronounced (Table 2). When the mutually exclusive diagnostic groups were considered, an early maternal diagnosis of anemia was associated with increased risk of ADHD (without comorbidities) (OR, 1.34; 95% CI, 1.08-1.66), ID without ASD (OR, 3.57; 95% CI, 2.41-5.27), and ASD with ID (OR, 2.23; 95% CI, 1.36-3.64) (Table 2). Risk for ASD (without comorbidities) was increased among offspring to mothers with later diagnosed anemia (OR, 1.13; 95% CI, 1.02-1.25). In continuous analysis, a pattern of decreasing risk with later gestational week at anemia diagnosis was observed for all outcomes except ASD with ADHD (Figure 2).

In adjusted models, adding socioeconomic and maternal- and pregnancy-related factors in combination attenuated the associations between maternal anemia and the outcomes (Table 2). After adjustment, we observed the strongest association between early (≤30 weeks) diagnosis of anemia and ID without co-occurring ASD (OR, 2.72; 95% CI, 1.84-4.01). Although the distribution of the covariates varied among the exposed and unexposed groups (Table 1) and all were associated with risk of neurodevelopmental disorders (eFigure 1), the modulating effect of any individual covariate was not extensive (eFigure 5 in the Supplement).

### Sensitivity Analysis

After stratification by birth year, the associations remained largely similar (eTables 5-7 in the Supplement), with ORs that were often lower among those born before 1997. An exception to this was ASD with ADHD, which was associated with maternal anemia only among those born before 1997 (OR, 1.28; 95% CI, 1.04-1.57) (eTables 5-7 in the Supplement).

### Sibling Analysis

Although there were a limited number of siblings exposed to maternal anemia diagnosed early in pregnancy, early anemia diagnosis was associated with increased risk for ASD and ID in the sibling analysis, with a higher OR for ASD (OR, 2.25; 95% CI, 1.24-4.11) compared with the primary analysis and a similar OR for ID (OR, 2.59; 95% CI, 1.08-6.22) (Table 2). The risk estimate for ADHD was similar to the primary analysis but with a wide CI (OR, 1.18; 95% CI, 0.79-1.76).

### Mediation Analysis

We evaluated as potential mediators obstetric complications that were associated with the outcomes of ASD, ADHD, or ID and with anemia diagnosed earlier in pregnancy (Table 1 and eFigure 4 and eFigure 6 in the Supplement) because only anemia diagnosed at 30 weeks or less was consistently associated with the outcomes. Adverse obstetric outcomes accounted for a modest proportion (2.2%-43.1%) of the association between maternal anemia and risk of ASD, ADHD, and ID, although the natural indirect relationship estimates were not statistically significant in most cases (Table 3). Excluding individuals born before 1997 yielded similar results. Preterm birth was the strongest mediator for all outcomes, particularly induced preterm birth, which accounted for approximately one-third of the association between anemia diagnosed at 30 weeks or less and risk of ASD (proportion mediated, 28.21%), ADHD (proportion mediated, 32.93%), and ID (proportion mediated, 32.03%).

**Table 3.** Mediation Analysis With Adverse Obstetric Outcomes as Potential Mediators Between Earlier Maternal Anemia (≤30 Weeks) and Offspring Risk of ASD, ADHD, and ID

Variable	Offspring, Odds Ratio (95% CI)^a^		
	Any ASD	Any ADHD	Any ID
**Early Anemia Diagnosis (≤30 wk), 1987-2010**			
Cesarean delivery			
Natural direct relationship	1.35 (1.00-1.80)	1.28 (1.02-1.55)	1.95 (1.29-2.77)
Natural indirect relationship	1.03 (0.95-1.14)	1.04 (0.98-1.12)	1.02 (0.91-1.16)
Total relationship	1.39 (1.06-1.82)	1.33 (1.07-1.58)	1.98 (1.32-2.77)
Proportion, %^b^	9.52	12.78	2.21
Low Apgar score			
Natural direct relationship	1.39 (1.05-1.82)	1.33 (1.07-1.60)	1.81 (1.22-2.55)
Natural indirect relationship	1.00 (0.98-1.04)	1.00 (0.98-1.04)	1.11 (1.00-1.31)
Total relationship	1.39 (1.05-1.81)	1.34 (1.08-1.61)	2.00 (1.31-2.82)
Proportion, %^b^	NR^c^	NR ^c^	14.81
Small for gestational age			
Natural direct relationship	1.45 (1.10-1.90)	1.35 (1.09-1.62)	1.92 (1.26-2.73)
Natural indirect relationship	1.00 (0.97-1.05)	1.01 (0.98-1.04)	1.07 (1.00-1.21)
Total relationship	1.45 (1.10-1.90)	1.36 (1.08-1.61)	2.05 (1.35-2.89)
Proportion, %^b^	NR^c^	1.90	9.27
Preterm (induced)			
Natural direct relationship	1.15 (0.75-1.53)	1.18 (0.93-1.48)	1.75 (1.08-2.58)
Natural indirect relationship	1.11 (0.77-1.35)	1.08 (0.99-1.21)	1.09 (0.93-1.43)
Total relationship	1.29 (0.94-1.67)	1.27 (1.03-1.56)	1.90 (1.22-2.75)
Proportion, %^b^	43.10	32.79	13.05
Preterm (spontaneous)			
Natural direct relationship	1.17 (0.80-1.55)	1.18 (0.93-1.47)	1.78 (1.08-2.57)
Natural indirect relationship	1.06 (0.98-1.21)	1.02 (0.97-1.11)	1.07 (0.97-1.27)
Total relationship	1.24 (0.88-1.65)	1.20 (0.95-1.50)	1.90 (1.17-2.75)
Proportion, %^b^	28.13	11.10	10.21
Preeclampsia			
Natural direct relationship	1.30 (0.95 -1.64)	1.27 (1.06-1.54)	1.86 (1.27-2.62)
Natural indirect relationship	1.04 (0.99-1.12)	1.01 (0.98-1.05)	1.07 (0.99-1.23)
Total relationship	1.35 (1.02-1.72)	1.27 (1.06-1.52)	1.99 (1.32-2.80)
Proportion, %^b^	13.1	2.45	9.39
**Early Anemia Diagnosis (≤30 wk), 1997-2010**			
Cesarean delivery			
Natural direct relationship	1.55 (1.06-2.09)	1.32 (0.99-1.66)	2.05 (1.15-3.01)
Natural indirect relationship	1.08 (0.96-1.28)	1.09 (0.98-1.21)	1.07 (0.92-1.32)
Total relationship	1.68 (1.22-2.22)	1.44 (1.09-1.77)	2.21 (1.31-3.19)
Proportion, %^b^	15.56	22.86	9.11
Low Apgar score			
Natural direct relationship	1.64 (1.14-2.18)	1.43 (1.07-1.77)	1.89 (1.07-2.83)
Natural indirect relationship	1.00 (0.97-1.04)	1.01 (0.98-1.07)	1.19 (1.02-1.53)
Total relationship	1.63 (1.17-2.15)	1.44 (1.07-1.76)	2.24 (1.29-3.23)
Proportion, %^b^	NR^c^	3.43	21.20
Small for gestational age			
Natural direct relationship	1.70 (1.23-2.28)	1.39 (1.05-1.72)	2.04 (1.18-3.01)
Natural indirect relationship	0.99 (0.97-1.04)	1.02 (0.98-1.08)	1.08 (1.00-1.27)
Total relationship	1.69 (1.22-2.23)	1.42 (1.06-1.74)	2.21 (1.31-3.18)
Proportion, %^b^	NR^c^	5.55	10.12
Preterm (induced)			
Natural direct relationship	1.43 (0.95-2.02)	1.27 (0.92-1.65)	1.65 (0.80-2.70)
Natural indirect relationship	1.15 (0.98-1.45)	1.13 (0.99-1.34)	1.27 (0.96-1.87)
Total relationship	1.64 (1.17-2.24)	1.43 (1.09-1.79)	2.10 (1.22-3.23)
Proportion, %^b^	28.21	32.93	32.03
Preterm (spontaneous)			
Natural direct relationship	1.45 (0.98-2.05)	1.28 (0.93-1.66)	1.74 (0.90-2.81)
Natural indirect relationship	1.07 (0.98-1.26)	1.05 (0.98-1.17)	1.20 (1.00-1.70)
Total relationship	1.55 (1.07-2.16)	1.34 (0.98-1.73)	2.09 (1.19-3.39)
Proportion, %^b^	15.90	15.22	24.53
Preeclampsia			
Natural direct relationship	1.63 (1.16-2.17)	1.46 (1.12-1.83)	2.05 (1.28.3.09)
Natural indirect relationship	1.03 (0.97-1.12)	1.01 (0.97-1.08)	1.12 (0.99-1.36)
Total relationship	1.68 (1.21-2.22)	1.47 (1.13-1.81)	2.39 (1.48-3.43)
Proportion, %^b^	6.30	2.00	13.68

Abbreviations: ADHD, attention-deficit/hyperactivity disorder; ASD, autism spectrum disorder; ID, intellectual disability; NR, not reported.

^a^ Adjusted for sex, birth year, educational level, disposable income, mother born outside Sweden, body mass index, maternal age, maternal psychiatric history, interpregnancy interval, and maternal infection during pregnancy.

^b^ Proportion mediated was calculated as log(natural indirect relationship)/log(total relationship).

^c^ The natural direct and natural indirect relationship were not in the same direction; therefore, the proportion mediated is not a logical value.

## Discussion

Although less prevalent, earlier diagnosed anemia was associated with a greater risk of ASD, ADHD, and ID compared with no diagnosis of anemia, even in models accounting for potentially confounding socioeconomic, maternal, and pregnancy-related factors. Anemia that occurred earlier in pregnancy was most strongly associated with the outcome of ID. The associations between anemia earlier in pregnancy and risk of ASD and ID were also apparent in matched sibling analyses. Obstetric complications previously associated with maternal anemia mediated a modest proportion of the risk of the neurodevelopmental disorders associated with earlier maternal anemia diagnosis.

### Agreement With Other Studies

Long-term cognitive and behavioral effects of maternal anemia have not been well studied in humans, especially regarding ASD and ADHD. Leonard et al^24^ found a 5-fold increased risk of severe ID in offspring of anemic mothers, although no increased risk for mild to moderate ID or ID with ASD was observed. In a secondary analysis of the Collaborative Perinatal Project, a dose-response relationship between maternal hematocrit during pregnancy and children’s IQ at 4 and 7 years of age was found.^25^ Women with moderate anemia had a 59% greater chance of having a child with an IQ below 70 at 7 years of age.^25^ To our knowledge, no other studies have been performed regarding maternal anemia during pregnancy and offspring risk of ASD, ADHD, and ID. A few small studies have found an increased risk of ASD associated with anemia in the infant,^26^ which can be the result of maternal anemia.^3^

Comparing the results of previous supplementation studies^5,6^ with our results warrants caution. Anemia status cannot be extrapolated from the supplementation studies, and we have not studied the effects of supplemental iron intake. Our results would support a potentially protective role of iron supplementation in pregnant women with regard to offspring risk of neurodevelopmental disorders^5^ because iron supplementation can prevent iron deficiency anemia.^21^ Regardless of neurodevelopmental outcomes, iron supplementation in pregnant women is associated with reduced risk of low birth weight and preterm birth.^21,27^ However, excessive iron intake can be toxic.^28,29^

### Interpretation and Potential Mechanisms

There is evidence that fetal needs for iron are prioritized compared with the mother’s.^30^ The fetus is not necessarily exposed to iron deficiency if the mother is affected by anemia but may be affected only when a threshold is crossed and the maternal shortage is more severe and long lasting.^3^ Although later anemia diagnoses may result from the higher iron demands of larger fetuses, early nutritional deficiencies represent a distinct phenomenon, leading to growth restriction and increased risk of being small for gestational age.^21^ Earlier anemia diagnoses were associated with infants being born small for gestational age, and later anemia diagnoses were associated with infants being born large for gestational age. The highest rates of fetal iron uptake occur from 30 weeks onward,^31^ with the neonate’s total iron endowment directly proportional to its birth weight.^32^ An alternative explanation could be that earlier diagnosed anemia occurs during a more critical time window with regard to neurodevelopmental disorders.

The associations reported here could be the result of iron deficiency in the developing brain. Iron is necessary for a number of developmental processes, such as myelination and dendrite arborization,^4^ and for the synthesis of monoamine neurotransmitters,^4^ which are implicated in the etiology of ASD and ADHD.^33,34^ Because erythrocyte hemoglobin is essential for the transport of oxygen, oxygen supply to the developing fetus might be limited in anemic mothers and may be associated with an increased risk of hypoxia. Alternatively, adverse obstetric outcomes caused by maternal anemia^21^ might mediate the association. Mediation analysis indicated that some adverse obstetric outcomes, particularly being born preterm, could explain a portion of the associations reported here.

### Strengths and Limitations

Strengths of our study include data that were prospectively collected in high-quality population-based registers from a setting with universal access to comprehensive health care, reducing bias in ascertainment of the exposure and outcomes. However, generalizability may be limited by the inclusion of only Swedish-born individuals. Another strength of this study is the consideration of multiple commonly co-occurring neurodevelopmental disorders as outcomes. For some diagnostic groups, we had a limited number of cases, especially when considering earlier anemia and when performing the mediation and sibling analysis. Also, this study integrated evidence from the sibling comparison with evidence from the multivariable regression models to address the issue of residual confounding.^35,36^ The sibling analysis accounted for factors that are largely shared by siblings, such as socioeconomic status and genetic background.

This study has limitations. Although we evaluated multiple covariates and include a sibling comparison, a possibility exists for residual confounding, for instance by other dietary deficiencies associated with anemia. Both obesity and being underweight may indicate a suboptimal diet. Adjusting for maternal BMI did not modulate the association between maternal anemia and offspring risk of neurodevelopmental disorders. Diet quality is associated with socioeconomic factors, which were also investigated as potential confounders.^37^ We were limited in our ascertainment of infections during pregnancy.^38^ Most infections do not require hospitalization, and we were not able to specifically examine infections known to be detrimental to the developing nervous system (eg, TORCH [*Toxoplasma gondii*, other, rubella virus, cytomegalovirus, and herpes simplex virus]).^39^ In addition, anemia caused by iron deficiency could not be disentangled from other causes. Given that most anemia is caused by iron deficiency,^40^ iron deficiency during pregnancy is the most plausible explanation of our results, although anemia regardless of its cause may affect neurodevelopment. Timing of anemia was determined by the first date of anemia diagnosis during or shortly before pregnancy. The true onset of micronutrient deficiencies and subsequent duration of anemia cannot be identified. Treatment of anemia caused by micronutrient deficiencies consists of supplying the necessary micronutrient, often via oral supplements but sometimes intravenously.^41^ Oral iron therapy can improve hemoglobin levels, although such therapy may not fully replete iron stores in pregnant women.^42^ Timing and effectiveness of treatment were unknown in our study.

## Conclusions

In this study, anemia diagnosed at 30 weeks or less of pregnancy was associated with modestly increased offspring risk of ASD and ADHD and greater risk of ID, suggesting that exposure to anemia earlier in gestation may be negatively associated with neurodevelopment in the child. Given that iron deficiency and anemia are common among women of childbearing age, our findings appear to emphasize the importance of early screening for iron status and nutritional counseling in antenatal care.

## Notes

Supplement. eTable 1. A Comparison of Individuals Included in the Final Study Sample to Those Excluded From the Study Sample eTable 2. Diagnostic Codes and Register Databases Used to Ascertain Diagnoses in the Stockholm Youth Cohort (SYC) eTable 3. A Comparison of Maternal and Child Characteristics for 1286 Women for Whom Gestational Age at Anemia Diagnosis Could Be Determined to 29732 Women for Whom Gestational Age Anemia Diagnosis Could Be Determined eTable 4. Prevalence and Odds Ratios (+95% Confidence Intervals) of Maternal Anemia Diagnosed During Pregnancy (in General and Diagnosed ≤30 Weeks or Diagnosed >30 Weeks) by Selected Characteristics eTable 5. Prevalence of Selected Characteristics and Pregnancy Outcomes of the Child by Anemia in a Cohort of Non-Adoptive Births in Sweden Between 1997 & 2010 eTable 6. Prevalence of Selected Characteristics and Pregnancy Outcomes of the Child by Anemia in a Cohort of Non-Adoptive Births in Sweden Between 1987 & 1996 eTable 7. Odds Ratios for ASD, ADHD, and ID in Offspring of Mothers Diagnosed With Anemia During Pregnancy After Stratification on Birth Years Before and After 1997 eFigure 1. The Association Between Potentially Confounding Factors and Risk of ASD, ADHD, or ID eFigure 2. Prevalence of Maternal Anemia per Birth Year eFigure 3. Prevalence of Maternal Anemia per Gestational Week eFigure 4. Risk for Pregnancy Outcomes in Relation to Diagnosis of Maternal Anemia, Comparing Mothers Diagnosed With Anemia During Pregnancy (at Any Point, Diagnosed ≤30 Weeks, or Diagnosed >30 Weeks) to Mothers Not Diagnosed With Anemia eFigure 5. An Exploration of the Influence of Different Potentially Confounding Factors of the Risk for Diagnostic Outcomes Related to Any Anemia Diagnosis, Anemia Diagnosed ≤30 Weeks and Anemia Diagnosed >30 Weeks eFigure 6. Odds Ratios (+95% Confidence Intervals) for Neurodevelopmental Disorders (ASD, ADHD, and ID) in Relation to Potential Mediators
