# Supplementary material for: Association of Prenatal Maternal Anemia With Neurodevelopmental Disorders
Source: JAMA Psychiatry. 2019 Sep 18;76(12):1294–304. doi: 10.1001/jamapsychiatry.2019.2309 (PMC6751782; doi:10.1001/jamapsychiatry.2019.2309)
Supplement: Supplement. — eTable 1. A Comparison of Individuals Included in the Final Study Sample to Those Excluded From the Study Sample eTable 2. Diagnostic Codes and Register Databases Used to Ascertain Diagnoses in the Stockholm Youth Cohort (SYC) eTable 3. A Comparison of Maternal and Child Characteristics for 1286 Women for Whom Gestational Age at Anemia Diagnosis Could Be Determined to 29732 Women for Whom Gestational Age Anemia Diagnosis Could Be Determined eTable 4. Prevalence and Odds Ratios (+95% Confidence Intervals) of Maternal Anemia Diagnosed During Pregnancy (in General and Diagnosed ≤30 Weeks or Diagnosed >30 Weeks) by Selected Characteristics eTable 5. Prevalence of Selected Characteristics and Pregnancy Outcomes of the Child by Anemia in a Cohort of Non-Adoptive Births in Sweden Between 1997 & 2010 eTable 6. Prevalence of Selected Characteristics and Pregnancy Outcomes of the Child by Anemia in a Cohort of Non-Adoptive Births in Sweden Between 1987 & 1996 eTable 7. Odds Ratios for ASD, ADHD, and ID in Offspring of Mothers Diagnosed With Anemia During Pregnancy After Stratification on Birth Years Before and After 1997 eFigure 1. The Association Between Potentially Confounding Factors and Risk of ASD, ADHD, or ID eFigure 2. Prevalence of Maternal Anemia per Birth Year eFigure 3. Prevalence of Maternal Anemia per Gestational Week eFigure 4. Risk for Pregnancy Outcomes in Relation to Diagnosis of Maternal Anemia, Comparing Mothers Diagnosed With Anemia During Pregnancy (at Any Point, Diagnosed ≤30 Weeks, or Diagnosed >30 Weeks) to Mothers Not Diagnosed With Anemia eFigure 5. An Exploration of the Influence of Different Potentially Confounding Factors of the Risk for Diagnostic Outcomes Related to Any Anemia Diagnosis, Anemia Diagnosed ≤30 Weeks and Anemia Diagnosed >30 Weeks eFigure 6. Odds Ratios (+95% Confidence Intervals) for Neurodevelopmental Disorders (ASD, ADHD, and ID) in Relation to Potential Mediators [file jamapsychiatry-76-1294-s001.pdf]

Supplementary Online Content

Wiegersma AM, Dalman C, Lee BK, Karlsson H, Gardner RM. Association of prenatal anemia and neurodevelopmental disorders. *JAMA Psychiatry.* Published online September 18, 2019. doi:10.1001/jamapsychiatry.2019.2309

eTable 1. A Comparison of Individuals Included in the Final Study Sample to Those Excluded From the Study Sample

eTable 2. Diagnostic Codes and Register Databases Used to Ascertain Diagnoses in the Stockholm Youth Cohort (SYC)

eTable 3. A Comparison of Maternal and Child Characteristics for 1286 Women for Whom Gestational Age at Anemia Diagnosis Could Not Be Determined to 29732 Women for Whom Gestational Age at Anemia Diagnosis Could Be Determined

eTable 4. Prevalence and Odds Ratios (+95% Confidence Intervals) of Maternal Anemia Diagnosed During Pregnancy (in General and Diagnosed ≤30 Weeks or Diagnosed >30 Weeks) by Selected Characteristics

eTable 5. Prevalence of Selected Characteristics and Pregnancy Outcomes of the Child by Anemia in a Cohort of Non-Adoptive Births in Sweden Between 1997 & 2010

eTable 6. Prevalence of Selected Characteristics and Pregnancy Outcomes of the Child by Anemia in a Cohort of Non-Adoptive Births in Sweden Between 1987 & 1996

eTable 7. Odds Ratios for ASD, ADHD, and ID in Offspring of Mothers Diagnosed With Anemia During Pregnancy After Stratification on Birth Years Before and After 1997

eFigure 1. The Association Between Potentially Confounding Factors and Risk of ASD, ADHD, or ID

eFigure 2. Prevalence of Maternal Anemia per Birth Year

eFigure 3. Prevalence of Maternal Anemia per Gestational Week

eFigure 4. Risk for Pregnancy Outcomes in Relation to Diagnosis of Maternal Anemia, Comparing Mothers Diagnosed With Anemia During Pregnancy (at Any Point, Diagnosed ≤30 Weeks, or Diagnosed >30 Weeks) to Mothers Not Diagnosed With Anemia

eFigure 5. An Exploration of the Influence of Different Potentially Confounding Factors of the Risk for Diagnostic Outcomes Related to Any Anemia Diagnosis, Anemia Diagnosed ≤30 Weeks and Anemia Diagnosed >30 Weeks

eFigure 6. Odds Ratios (+95% Confidence Intervals) for Neurodevelopmental Disorders (ASD, ADHD, and ID) in Relation to Potential Mediators

This supplementary material has been provided by the authors to give readers additional information about their work.

		Excluded in cohort	Included in cohort
**N**		203964	532232
**Mother born outside of Sweden**		90632 (47.5%)	132593 (24.9%)
**Index person born outside of Sweden**		72534 (35.6%)	0 (0.0%)
**Male**		104435 (51.2%)	272884 (51.3%)
**Maternal age at IP's birth**	<25	43870 (21.5%)	79113 (14.9%)
	25-29	57280 (28.1%)	156485 (29.4%)
	30-34	56603 (27.8%)	184375 (34.6%)
	35-39	27171 (13.3%)	92810 (17.4%)
	≥40	5873 (2.9%)	19449 (3.7%)
	Missing	13167 (6.5%)	0 (0.0%)
**Disposable Income at IP's birth, 5 quintiles**	1st	69055 (33.9%)	76068 (14.3%)
	2nd	32930 (16.1%)	112120 (21.1%)
	3rd	28889 (14.2%)	115649 (21.7%)
	4th	29463 (14.4%)	114727 (21.6%)
	5th	30428 (14.9%)	113668 (21.4%)
	Missing	13199 (6.5%)	0 (0.0%)
**Highest parental education level**	≤9 years	14960 (7.3%)	30613 (5.8%)
	10-12 years	52546 (25.8%)	202078 (38.0%)
	>12 years	94552 (46.4%)	290430 (54.6%)
	Missing	41906 (20.5%)	9111 (1.7%)
**Maternal psychiatric history before IP’s birth (Any diagnosis)**		53008 (27.8%)	172207 (32.4%)
**Multiple birth**		1758 (2.3%)	14572 (2.7%)

eTable 1. ***A comparison of individuals included in the final study sample to those excluded from the study sample.*** See Figure 1A in the main text for reasons for exclusion.

© 2019 Wiegersma AM et al. *JAMA Psychiatry.*

Coding system	Autism Spectrum Disorders (ASD)	Attention Deficit/Hyperactivity Disorder (ADHD)	Intellectual Disability	Maternal anemia	Parental psychiatric history
ICD-9[a]	299	314	317-319	648C[f] & 280[g]	290-319
ICD-10[b,c]	F84	F90	F70-F79	O99.0[f] & D50[g]	F chapter
DSM-IV[b]	299	314	317-319		
Other	Habilitation Register[d]	Prescription Drug Register[e]: methylphenidate [N06BA04] or atomoxetin [N06BA09]	Habilitation Register[d]		ICD8: 290-315

[a] The National Patient Register (NPR): including inpatient care from 1973, outpatient physician visits in specialist care from 1997.

[b] Stockholm Clinical Database for Child and Adolescent Psychiatry: DSM-IV was used until 2008; ICD-10 was used thereafter.

[c] VAL register: Stockholm county health care databases including in- and outpatient care, regardless of specialty/primary health care.

[d] The Habilitation Register (HAB): Provides data on utilization of Stockholm County Habilitation services according to type of disability, such as pervasive developmental disorders and intellectual disability (recorded as present or absent). HAB register was used until 2008; since 2008 it has been included in the VAL databases as ICD-coded diagnoses (see C above).

[e] The Prescription Drug Register (PDR) contains data on medications dispensed to the entire population in Sweden since 1 July 2005. Receipt of a prescription for ADHD medications is a useful proxy for an ADHD diagnosis, as Swedish medical guidelines mandate that ADHD medications should only be prescribed by a psychiatric specialist and after other (non-pharmacological) interventions have failed.

[f] Anemia complicating pregnancy

[g] Iron deficiency anemia

eTable 2. ***Diagnostic codes and register databases used to ascertain diagnoses in the Stockholm Youth Cohort (SYC).***

© 2019 Wiegersma AM et al. *JAMA Psychiatry.*

		Anemia diagnosis date unknown	Anemia diagnosis date known
**N**		1286	29732
**Mother born outside of Sweden**		404 (31.4%)	8473 (28.5%)
**Maternal age, years**	<25	179 (13.9%)	3754 (12.6%)
	25-29	357 (27.8%)	7888 (26.5%)
	30-34	425 (33.0%)	10763 (36.2%)
	35-39	247 (19.2%)	5888 (19.8%)
	≥40	78 (6.1%)	1439 (4.8%)
**Disposable Income at IP's birth, 5 quintiles**	1st	200 (15.6%)	4012 (13.5%)
	2nd	288 (22.4%)	6318 (21.2%)
	3rd	266 (20.7%)	6287 (21.1%)
	4th	259 (20.1%)	6475 (21.8%)
	5th	273 (21.2%)	6640 (22.3%)
**Highest parental education level**	≤9 years	89 (6.9%)	1510 (5.1%)
	10-12 years	453 (35.2%)	10546 (35.5%)
	>12 years	708 (55.1%)	17052 (57.4%)
	Missing	36 (2.8%)	624 (2.1%)
**Maternal psychiatric history before IP's birth (Any diagnosis)**		467 (36.3%)	10007 (33.7%)
**Maternal body mass index (kg/m^2)**	Normal	641 (49.8%)	15044 (50.6%)
	Underweight	32 (2.5%)	768 (2.6%)
	Overweight	228 (17.7%)	5351 (18.0%)
	Obese	89 (6.9%)	2081 (7.0%)
	Missing	296 (23.0%)	6488 (21.8%)
**Male**		708 (55.1%)	15414 (51.8%)
**Multiple birth**		184 (14.3%)	2135 (7.2%)
**Gestational age at birth**	Preterm	161 (12.8%)	2570 (8.6%)
	Term	932 (74.0%)	24254 (81.6%)
	Post-term	167 (13.3%)	2908 (9.8%)
**Size for gestational age**	Normal	987 (76.7%)	25333 (85.2%)
	SGA	36 (2.8%)	648 (2.2%)
	LGA	53 (4.1%)	1494 (5.0%)
	Missing	210 (16.3%)	2257 (7.6%)
**C-section birth**		439 (34.1%)	9994 (33.6%)
**Low Apgar score (<7)**		40 (3.1%)	507 (1.7%)

eTable 3. ***A comparison of maternal and child characteristics for 1286 women for whom gestational age at anemia diagnosis could not be determined to 29732 women for whom gestational age at anemia diagnosis could be determined.***

© 2019 Wiegersma AM et al. *JAMA Psychiatry.*

	No maternal anemia N (%)	Maternal Anemia N(%)	OR (95% CI)	Maternal Anemia ≤30weeks N(%)	OR (95% CI)	Maternal Anemia >30 weeks N(%)	OR (95% CI)
**Sex**							
Male	256 762 (51.23)	16 122 (51.98)	1.03 (1.01-1.05)	788 (51.37)	1.01 (0.91-1.11)	14 626 (51.87)	1.03 (1.00-1.05)
Female	244 452 (48.77)	14 896 (48.02)	Reference	746 (48.63)	Reference	13 572 (48.13)	Reference
**Maternal body mass index (kg/m^2)**							
Normal	252 748 (50.43)	15 685 (50.57)	Reference	725 (47.26)	Reference	14 319 (50.78)	Reference
Underweight	13 399 (2.67)	800 (2.58)	1.11 (1.03-1.19)	52 (3.39)	1.46 (1.09-1.96)	716 (2.54)	1.10 (1.01-1.18)
Overweight	71 039 (14.17)	5579 (17.99)	1.16 (1.12-1.19)	250 (16.30)	1.14 (0.98-1.32)	5101 (18.09)	1.16 (1.12-1.20)
Obese	23 675 (4.72)	2170 (7.00)	1.28 (1.22-1.34)	103 (6.71)	1.34 (1.08-1.65)	1978 (7.01)	1.28 (1.21-1.14)
Missing	140 353 (28.00)	6784 (21.87)	1.10 (1.07-1.14)	404 (26.34)	1.40 (1.23-1.59)	6084 (21.58)	1.10 (1.07-1.14)
**Maternal age**							
<25	75 180 (15.00)	3933 (12.68)	Reference	251 (16.36)	Reference	3503 (12.42)	Reference
25-29	148 240 (29.58)	8245 (26.58)	0.97 (0.93-1.00)	403 (26.27)	0.78 (0.66-0.91)	7485 (26.54)	0.98 (0.94-1.02)
30-34	173 187 (34.55)	11 188 (36.07)	0.97 (0.94-1.01)	501 (32.66)	0.75 (0.64-0.88)	10 262 (36.39)	0.99 (0.95-1.03)
35-39	86 675 (17.29)	6135 (19.78)	1.00 (0.96-1.04)	304 (19.82)	0.85 (0.71-1.01)	5584 (19.80)	1.01 (1.10-1.25)
≥40	17 932 (3.58)	1517 (4.89)	1.17 (1.10-1.25)	75 (4.89)	0.96 (0.74-1.26)	1364 (4.84)	1.17 (1.10-1.25)
**Disposable income at IP´s birth (5 quintiles)**							
1st	71 856 (14.34)	4212 (13.58)	Reference	315 (20.53)	Reference	3697 (13.11)	Reference
2nd	105 514 (21.05)	6606 (21.30)	0.98 (0.94-1.02)	429 (27.97)	0.87 (0.75-1.01)	5889 (20.88)	0.99 (0.95-1.03)
3rd	109 096 (21.77)	6553 (21.13)	0.93 (0.90-0.97)	324 (21.12)	0.64 (0.55-0.75)	5963 (21.15)	0.96 (0.92-1.00)
4th	107 993 (21.55)	6734 (21.71)	0.98 (0.94-1.02)	247 (16.10)	0.50 (0.42-0.60)	6228 (22.09)	1.03 (0.98-1.07)
5th	106 755 (21.30)	6913 (22.29)	1.04 (1.00-1.08)	219 (14.28)	0.46 (0.39-0.55)	6421 (22.77)	1.09 (1.04-1.14)
**Highest parental education level**							
≤9 years	29 014 (5.79)	1599 (5.16)	Reference	129 (8.41)	Reference	1381 (4.90)	Reference
10-12 years	191 079 (38.12)	10 999 (35.46)	0.99 (0.93-1.05)	643 (41.92)	0.74 (0.61-0.91)	9903 (35.12)	1.02 (0.96-1.09)
>12 years	272 670 (54.40)	17 760 (57.26)	0.96 (0.91-1.02)	727 (47.39)	0.49 (0.40-0.60)	16 325 (57.89)	1.01 (0.95-1.07)
Missing	8451 (1.69)	660 (2.13)	1.15 (1.04-1.27)	35 (2.28)	0.69 (0.47-1.02)	589 (2.09)	1.18 (1.06-1.31)
**Maternal psychiatric history before IP's birth (Any diagnosis)**							
Not present	355 244 (67.85)	20 544 (66.23)	Reference	908 (59.19)	Reference	18 817 (66.73)	Reference
Present	161 733 (32.27)	10 474 (33.77)	1.12 (1.09-1.15)	626 (40.81)	1.54 (1.38-1.72)	9381 (33.27)	1.10 (1.07-1.13)
**Single or multiple birth**							
Single birth	488 961 (97.56)	28 699 (92.52)	Reference	1360 (88.66)	Reference	26 237 (93.05)	Reference
Multiple birth	12 253 (2.44)	2319 (7.48)	3.09 (2.94-3.24)	174 (11.34)	5.04 (4.28-5.93)	1961 (6.95)	2.87 (2.72-3.02)
**Birth order (parity)**							

© 2019 Wiegersma AM et al. *JAMA Psychiatry.*

1st child	224 443 (44.78)	17 320 (55.84)	Reference	575 (37.48)	Reference	15 982 (56.68)	Reference
2nd child	183 821 (36.68)	8934 (28.80)	0.63 (0.61-0.65)	540 (35.20)	1.14 (1.02-1.27)	8074 (28.63)	0.62 (0.60-0.63)
3rd or later child	92 950 (18.54)	4764 (15.36)	0.68 (0.65-0.70)	419 (27.31)	1.73 (1.53-1.97)	4142 (14.69)	0.64 (0.62-0.66)
**Mother hospitalized for infection during pregnancy**							
No	483 099 (96.56)	28 619 (92.34)	Reference	1336 (87.09)	Reference	26 139 (92.70)	Reference
Yes	17 229 (3.44)	2373 (7.66)	2.04 (1.95-2.14)	198 (12.91)	3.62 (3.10-4.21)	2059 (7.30)	1.95 (1.85-2.04)
**Mother born in Sweden**							
Yes	377 498 (75.32)	22 141 (71.38)	Reference	862 (56.19)	Reference	20 397 (72.33)	Reference
No	123 716 (24.68)	8877 (28.62)	1.17 (1.14-1.20)	672 (43.81)	2.31 (2.07-2.57)	7801 (27.67)	1.12 (1.09-1.15)
**Interpregnancy interval**							
First born	224 443 (44.78)	17 320 (55.84)	1.57 (1.52-1.63)	575 (37.48)	0.78 (0.68-0.89)	15 982 (56.68)	1.62 (1.17-1.35)
<1 year	42 557 (8.49)	1941 (6.26)	0.98 (0.92-1.03)	157 (10.23)	1.10 (0.91-1.34)	1709 (6.06)	0.97 (0.91-1.02)
1-2 years	81 891 (16.34)	3579 (11.54)	0.91 (0.87-0.95)	194 (12.65)	0.73 (0.61-0.87)	3246 (11.51)	0.92 (0.87-0.96)
2-5 years	91 657 (18.29)	4500 (14.51)	Reference	307 (20.01)	Reference	4037 (14.32)	Reference
5-10 years	33 499 (6.68)	1871 (6.03)	1.15 (1.09-1.21)	141 (9.19)	1.29 (1.06-1.58)	1652 (5.86)	1.13 (1.07-1.20)
>10 years	8465 (1.69)	539 (1.74)	1.26 (1.15-1.38)	40 (2.61)	1.38 (0.99-1.92)	472 (1.67)	1.23 (1.11-1.36)
Missing	18 702 (3.73)	1268 (4.09)	1.30 (1.22-1.39)	120 (7.82)	1.76 (1.42-2.19)	1100 (3.90)	1.26 (1.17-1.35)

eTable 4. ***Prevalence and odds ratios (+95% confidence intervals) of maternal anemia diagnosed during pregnancy (in general and diagnosed ≤30 weeks or diagnosed >30 weeks) by selected characteristics.*** Odds ratios were calculated via generalized estimating equation (GEE) modeling with logit link clustered on maternal identification number.

© 2019 Wiegersma AM et al. *JAMA Psychiatry.*

	Maternal Anemia N(%)	No maternal anemia N(%)	Diagnosis ≤30 weeks N(%)	Diagnosis >30 weeks N(%)
**Total**	25 236	288 551	1136	23 292
**Offspring diagnosis**				
Unaffected	22 852 (90.55)	262 903 (91.11)	1000 (88.03)	21 146 (90.79)
Any ASD	929 (3.68)	9 762 (3.38)	55 (4.84)	831 (3.57)
Any ADHD	1801 (7.14)	19 911 (6.90)	101 (8.89)	1622 (6.96)
Any ID	274 (1.09)	2 658 (0.92)	29 (2.55)	230 (0.99)
ASD only	401 (1.59)	4 022 (1.39)	20 (1.76)	368 (1.58)
ADHD only	1 319 (5.23)	14 639 (5.07)	63 (5.55)	1204 (5.17)
ID without ASD	136 (0.54)	1247 (0.43)	18 (1.58)	111 (0.48)
ASD + ID (includes ASD + ID + ADHD)	138 (0.55)	1411 (0.49)	11 (0.97)	119 (0.51)
ASD + ADHD (excluding ASD + ID + ADHD)	390 (1.55)	4329 (1.50)	24 (2.11)	344 (1.48)
**Sex**				
Male	13 115 (51.97)	148 005 (51.29)	578 (50.88)	12 094 (51.92)
Female	12 121 (48.03)	140 546 (48.71)	558 (49.12)	11 198 (48.08)
**Maternal body mass index (kg/m^2)**				
Normal	13 114 (51.97)	161 650 (56.02)	573 (50.44)	12 119 (52.03)
Underweight	605 (2.40)	6 872 (2.38)	43 (3.79)	548 (2.35)
Overweight	4934 (19.55)	51 185 (17.74)	205 (18.05)	4562 (19.59)
Obese	2009 (7.96)	18 523 (6.42)	89 (7.83)	1844 (7.92)
Missing	4574 (18.12)	50 321 (17.44)	226 (19.89)	4219 (18.11)
**Maternal age (years)**				
<25	2731 (10.82)	31 934 (11.07)	174 (15.32)	2482 (10.66)
25-29	6345 (25.14)	74 653 (25.87)	279 (24.56)	5862 (25.17)
30-34	9439 (37.40)	109.679 (38.01)	363 (31.95)	8794 (37.76)
35-39	5388 (21.35)	59 807 (20.73)	248 (21.83)	4954 (21.27)
≥40	1333 (5.28)	12 478 (4.32)	72 (6.34)	1200 (5.15)
**Disposable income at IP´s birth (5 quintiles)**				
1st	3311 (13.12)	36 719 (12.73)	240 (21.13)	2966 (12.73)
2nd	5440 (21.56)	61 912 (21.46)	323 (28.43)	4937 (21.20)
3th	5494 (21.77)	64 764 (22.44)	249 (21.92)	5064 (21.74)
4th	5510 (21.83)	63 561 (22.03)	171 (15.05)	5174 (22.21)
5th	5481 (21.72)	61 595 (21.35)	153 (13.47)	5151 (22.11)
**Highest parental education level**				
≤9 years	1189 (4.71)	13 103 (4.54)	91 (8.01)	1054 (4.53)
10-12 years	8451 (33.49)	94 766 (32.84)	434 (38.20)	7767 (33.35)
>12 years	15 019 (59.51)	175 266 (60.74)	578 (50.88)	13 950 (59.89)
Missing	577 (2.29)	5 416 (1.88)	33 (2.90)	521 (2.24)
**Maternal psychiatric history before IP’s birth (Any diagnosis)**				
Not present	16 791 (66.54)	200 924 (69.63)	673 (59.24)	15 616 (67.04)
Present	8445 (33.46)	87 627 (30.37)	463 (40.76)	7676 (32.96)
**Single or multiple birth**				
Single birth	23 224 (92.03)	281 350 (97.50)	990 (87.15)	21 593 (92.71)
Multiple birth	2012 (7.97)	7 201 (2.50)	146 (12.85)	1699 (7.29)
**Birth order (parity)**				
1st child	14 235 (56.41)	130 041 (45.07)	417 (36.71)	13 308 (57.14)
2nd child	7212 (28.58)	107 005 (37.08)	386 (33.98)	6640 (28.51)
3rd or later child	3789 (15.01)	51 505 (17.85)	333 (29.31)	3344 (14.36)
**Mother hospitalized for infection during pregnancy**				
No	23 154 (91.81)	276 422 (95.91)	968 (85.21)	21 491 (92.27)
Yes	2065 (8.19)	11 791 (4.09)	168 (14.79)	1801 (7.73)
**Mother born in Sweden**				
Yes	17 750 (70.34)	213 344 (73.94)	588 (51.76)	16 616 (71.34)
No	7486 (29.66)	75 207 (26.06)	548 (48.24)	6676 (28.66)
**Interpregnancy interval**				

© 2019 Wiegersma AM et al. *JAMA Psychiatry.*

First born	14 235 (56.41)	130 041 (45.07)	417 (36.71)	13 308 (57.14)
<1 year	1482 (5.87)	22 242 (7.71)	125 (11.00)	1318 (5.66)
1-2 years	2820 (11.17)	45 748 (15.85)	138 (12.15)	2615 (11.23)
2-5 years	3619 (14.34)	54 147 (18.77)	226 (19.89)	3311 (14.22)
5-10 years	1564 (6.20)	19 719 (6.83)	101 (8.89)	1409 (6.05)
>10 years	456 (1.81)	5043 (1.75)	28 (2.46)	408 (1.75)
Missing	1060 (4.20)	11 611 (4.02)	101 (8.89)	923 (3.96)
**Size for gestational age**				
Small for gestational age	545 (2.16)	5 950 (2.06)	75 (6.60)	450 (1.93)
Normal	21 269 (84.28)	265 425 (91.99)	880 (77.46)	19 829 (85.13)
Large for gestational age	1288 (5.10)	8808 (3.05)	30 (2.64)	1214 (5.21)
Missing size for gestational age	122 (0.48)	1167 (0.40)	5 (0.44)	100 (0.43)
Missing due to multiple birth	2012 (7.97)	7201 (2.50)	146 (12.85)	1699 (7.29)
**Low Apgar score (<7)**				
No	24 556 (98.12)	284 019 (99.18)	1066 (94.92)	22 727 (98.35)
Yes	471 (1.88)	2348 (0.82)	57 (5.08)	381 (1.65)
**C-section at birth**				
No	16 208 (64.23)	236 398 (81.93)	657 (57.83)	15 107 (64.86)
Yes	9028 (35.77)	52 144 (18.07)	479 (42.17)	8185 (35.14)
**Gestational age at birth**				
Preterm	2303 (9.13)	15 021 (5.21)	399 (35.12)	1763 (7.57)
Term	20 421 (80.97)	254 220 (88.21)	687 (60.48)	19 195 (82.41)
Post-term	2495 (9.89)	18 972 (6.58)	50 (4.40)	2334 (10.02)

eTable 5. ***Prevalence of selected characteristics and pregnancy outcomes of the child by anemia in a cohort of non-adoptive births in Sweden between 1997 & 2010.***

© 2019 Wiegersma AM et al. *JAMA Psychiatry.*

	Maternal Anemia N(%)	No maternal anemia N(%)	Diagnosis ≤30 weeks N(%)	Diagnosis >30 weeks N(%)
**Total**	5782	212 663	398	4906
**Offspring diagnosis**				
Unaffected	5191 (89.78)	192 837 (90.68)	347 (87.19)	4418 (90.05)
Any ASD	218 (3.77)	6761 (3.18)	14 (3.52)	183 (3.73)
Any ADHD	451 (7.80)	14 979 (7.04)	37 (9.30)	375 (7.64)
Any ID	87 (1.50)	3246 (1.53)	14 (3.52)	66 (1.35)
ASD only	80 (1.38)	2710 (1.27)	6 (1.51)	67 (1.37)
ADHD only	329 (5.69)	11 229 (5.28)	28 (7.04)	274 (5.58)
ID without ASD	44 (0.76)	1836 (0.86)	9 (2.26)	31 (0.63)
ASD + ID (includes ASD + ID + ADHD)	43 (0.74)	1410 (0.66)	5 (1.26)	35 (0.71)
ASD + ADHD (excluding ASD + ID + ADHD)	95 (1.64)	2641 (1.24)	3 (0.75)	81 (1.65)
**Sex**				
Male	3007 (52.01)	108 757 (51.14)	210 (52.76)	2532 (51.61)
Female	2775 (47.99)	103 906 (48.86)	188 (47.24)	2374 (48.39)
**Maternal body mass index (kg/m^2)**				
Normal	2571 (44 47)	91 098 (42 84)	152 (38.19)	2200 (44.84)
Underweight	195 (3.37)	6527 (3 07)	9 (2.26)	168 (3.42)
Overweight	645 (11.16)	19 854 (9.34)	45 (11.31)	539 (10.99)
Obese	161 (2.78)	5152 (2.42)	14 (3.52)	134 (2.73)
Missing	2210 (38.22)	90 032 (42.34)	178 (44.72)	1865 (38.01)
**Maternal age (years)**				
<25	1202 (20.79)	43 246 (20.34)	77 (19.35)	1021 (20.81)
25-29	1900 (32.86)	73 587 (34.60)	124 (31.16)	1623 (33.08)
30-34	1749 (30.25)	63 508 (29.86)	138 (34.67)	1468 (29.92)
35-39	747 (12.92)	26 868 (12.63)	56 (14.07)	630 (12.84)
≥40	184 (3.18)	5454 (2.56)	3 (0.75)	164 (3.34)
**Disposable income at IP´s birth (5 quintiles)**				
1st	901 (15.58)	35 137 (16.52)	75 (18.84)	731 (14.90)
2nd	1166 (20.17)	43 602 (20.50)	106 (26.63)	952 (19.40)
3th	1059 (18.32)	44 332 (20.85)	75 (18.84)	899 (18.32)
4th	1224 (21.17)	44 432 (20.89)	76 (19.10)	1054 (21.48)
5th	1432 (24.77)	45 160 (21.24)	66 (16.58)	1270 (25.89)
**Highest parental education level**				
≤9 years	410 (7.09)	15 911 (7.48)	38 (9.55)	327 (6.67)
10-12 years	2548 (44.07)	96 313 (45.29)	209 (52.51)	2136 (43.54)
>12 years	2741 (47.41)	97 404 (45.80)	149 (37.44)	2375 (48.41)
Missing	83 (1.44)	3035 (1.43)	2 (0.50)	68 (1.39)
**Maternal psychiatric history before IP's birth (Any diagnosis)**				
Not present	3753 (64.91)	138 557 (65.15)	235 (59.05)	3201 (65.25)
Present	2029 (35.09)	74 106 (34.85)	163 (40.95)	1705 (34.75)
**Single or multiple birth**				
Single birth	5475 (94.69)	207 611 (97.62)	370 (92.96)	4644 (94.66)
Multiple birth	5052 (2.38)	307 (5.31)	28 (7.04)	262 (5.34)
**Birth order (parity)**				
1st child	3085 (53.36)	94 402 (44.39)	158 (39.70)	2674 (54.50)
2nd child	1722 (29.78)	76 816 (36.12)	154 (38.69)	1434 (29.23)
3rd or later child	975 (16.86)	41 445 (19.49)	86 (21.61)	798 (16.27)
**Mother hospitalized for infection during pregnancy**				
No	5465 (94.66)	206 677 (97.44)	368 (92.46)	4648 (94.74)
Yes	308 (5.34)	5438 (2.56)	30 (7.54)	258 (5.26)
**Mother born in Sweden**				
Yes	4391 (75.94)	164 154 (77.19)	274 (68.84)	3781 (77.07)
No	1391 (24.06)	48 509 (22.81)	124 (31.16)	1125 (22.93)
**Interpregnancy interval**				

© 2019 Wiegersma AM et al. *JAMA Psychiatry.*

First born	3085 (53.36)	94 402 (44.39)	158 (39.70)	2674 (54.50)
<1 year	459 (7.94)	20 315 (9.55)	32 (8.04)	391 (7.97)
1-2 years	759 (13.13)	36 143 (17.00)	56 (14.07)	631 (12.86)
2-5 years	881 (15.24)	37 510 (17.64)	81 (20.35)	726 (14.80)
5-10 years	307 (5.31)	13 780 (6.48)	40 (10.05)	243 (4.95)
>10 years	83 (1.44)	3422 (1.61)	12 (3.02)	64 (1.30)
Missing	208 (3.60)	7091 (3.33)	19 (4.77)	177 (3.61)
**Size for gestational age**				
Small for gestational age	139 (2.40)	5811 (2.73)	17 (4.27)	106 (2.16)
Normal	5051 (87.36)	195 000 (91.69)	332 (83.42)	4292 (87.48)
Large for gestational age	259 (4.48)	5509 (2.59)	17 (4.27)	233 (4.75)
Missing size for gestational age	26 (0.45)	1291 (0.61)	4 (1.01)	13 (0.27)
Missing due to multiple birth	307 (5.31)	5052 (2.37)	28 (7.03)	262 (5.34)
**Low Apgar score (<7)**				
No	5672 (98.68)	208 978 (99.04)	378 (97.97)	4816 (98.75)
Yes	76 (1.32)	2015 (0.96)	8 (2.03)	61 (1.25)
**C-section at birth**				
No	4377 (75.70)	186 582 (87.74)	299 (75.13)	3675 (74.91)
Yes	1405 (24.30)	26 081 (12.26)	99 (24.87)	1231 (25.09)
**Gestational age at birth**				
Preterm	428 (7.41)	11 825 (5.57)	96 (24.12)	312 (6.36)
Term	4765 (82.54)	183 644 (86.58)	286 (71.86)	4086 (83.29)
Post-term	580 (10.05)	16 646 (7.85)	16 (4.02)	508 (10.35)

eTable 6. ***Prevalence of selected characteristics and pregnancy outcomes of the child by anemia in a cohort of non-adoptive births in Sweden between 1987 & 1996.***

© 2019 Wiegersma AM et al. *JAMA Psychiatry.*

	< 1997			≥ 1997		
	Anemia (all prenatal diagnoses)					
	N (%)	Model 1 OR (95%CI)[a]	Model 2 OR (95%CI)[b]	N (%)	Model 1 OR (95%CI)[c]	Model 2 OR (95%CI)[d]
**Any ASD**	218 (4.03)	1.15 (1.00-1.32)	1.14 (0.99-1.31)	929 (3.91)	1.10 (1.03-1.18)	1.04 (0.97-1.11)
**Any ADHD**	451 (7.99)	1.08 (0.98-1.19)	1.09 (0.99-1.21)	1801 (7.31)	1.06 (1.01-1.11)	1.03 (0.97-1.08)
**Any ID**	87 (1.65)	0.98 (0.79-1.21)	0.97 (0.78-1.21)	274 (1.18)	1.20 (1.06-1.36)	1.09 (0.96-1.24)
**ASD**	80 (1.52)	1.06 (0.85-1.33)	1.05 (0.84-1.31)	401 (1.72)	1.16 (1.04-1.28)	1.09 (0.98-1.21)
**ADHD**	329 (5.96)	1.06 (0.95-1.18)	1.07 (0.96-1.20)	1319 (5.46)	1.06 (1.00-1.12)	1.04 (0.98-1.10)
**ID without ASD**	44 (0.84)	0.89 (0.66-1.20)	0.89 (0.66-1.20)	136 (0.59)	1.29 (1.08-1.54)	1.15 (0.96-1.38)
**ASD + ID**	43 (0.82)	1.09 (0.80-1.48)	1.06 (0.78-1.44)	138 (0.60)	1.13 (0.94-1.34)	1.04 (0.87-1.24)
**ASD + ADHD**	95 (1.80)	1.27 (1.03-1.56)	1.28 (1.04-1.57)	390 (1.68)	1.05 (0.95-1.17)	0.99 (0.89-1.10)
	Anemia diagnosis ≤30 weeks					
**Any ASD**	14 (3.88)	1.10 (0.64-1.--87)	--[c]	55 (5.21)	1.75 (1.33-2.30)	1.61 (1.22-2.12)
**Any ADHD**	37 (9.64)	1.32 (0.94-1.85)	--[c]	101 (9.17)	1.58 (1.28-1.95)	1.45 (1.17-1.79)
**Any ID**	14 (3.88)	2.30 (1.34-3.94)	--[c]	29 (2.82)	3.25 (2.22-4.77)	2.27 (1.55-3.32
**ASD**	6 (1.70)	1.16 (0.51-2.63)	--[c]	20 (1.96)	1.49 (0.96-2.31)	1.44 (0.92-2.24)
**ADHD**	28 (7.47)	1.33 (0.90-1.95)	--[c]	63 (5.93)	1.36 (1.05-1.77)	1.23 (0.94-1.60)
**ID without ASD**	9 (2.53)	2.57 (1.30-5.06)	--[c]	18 (1.77)	4.44 (2.76-7.16)	2.96 (1.84-4.78)
**ASD + ID**	5 (1.42)	1.94 (0.81-4.64)	--[c]	11 (1.09)	2.38 (1.30-4.35)	1.71 (0.94-3.13)
**ASD + ADHD**	3 (0.86)	0.60 (0.19-1.87)	--[c]	24 (2.34)	1.80 (1.20-2.71)	1.71 (1.13-2.58)
	Anemia diagnosis >30 weeks					
**Any ASD**	183 (3.98)	1.13 (0.97-1.32)	--[c]	831 (3.78)	1.06 (0.99-1.14)	1.00 (0.93-1.07)
**Any ADHD**	375 (7.82)	1.06 (0.95-1.18)	--[c]	1622 (7.12)	1.02 (0.97-1.08)	0.99 (0.94-1.05)
**Any ID**	66 (1.47)	0.88 (0.69-1.12)	--[c]	230 (1.08)	1.08 (0.95-1.24)	1.00 (0.87-1.15)
**ASD**	67 (1.49)	1.05 (0.82-1.34)	--[c]	368 (1.71)	1.14 (1.03-1.27)	1.07 (0.96-1.20)
**ADHD**	274 (5.84)	1.04 (0.92-1.17)	--[c]	1204 (5.39)	1.04 (0.98-1.10)	1.02 (0.96-1.08)
**ID without ASD**	31 (0.70)	0.75 (0.52-1.06)	--[c]	111 (0.52)	1.13 (0.93-1.37)	1.03 (0.85-1.26)
**ASD + ID**	35 (0.79)	1.04 (0.74-1.46)	--[c]	119 (0.56)	1.05 (0.87-1.26)	0.98 (0.81-1.18)
**ASD + ADHD**	81 (1.80)	1.28 (1.02-1.60)	--[c]	344 (1.60)	1.00 (0.89-1.11)	0.94 (0.84-1.05)

[a] Model 1: Generalized estimating equation (GEE) model, clustered on maternal ID, adjusted only for birth year and sex.

[b] Model 2: Adjusted for: birth year, sex, education, disposable income, mother born outside Sweden, BMI, maternal age, maternal psychiatric history, multiple birth, interpregnancy interval and maternal infection during pregnancy.

[c] Due to the low number of cases in some groups after stratification for birth year and timing of diagnosis, we do not have sufficient power to adjust for all covariates in Model b.

eTable 7. Odds ratios for ASD, ADHD and ID in offspring of mothers diagnosed with anemia during pregnancy after stratification on birth years before and after 1997.

© 2019 Wiegersma AM et al. *JAMA Psychiatry.*

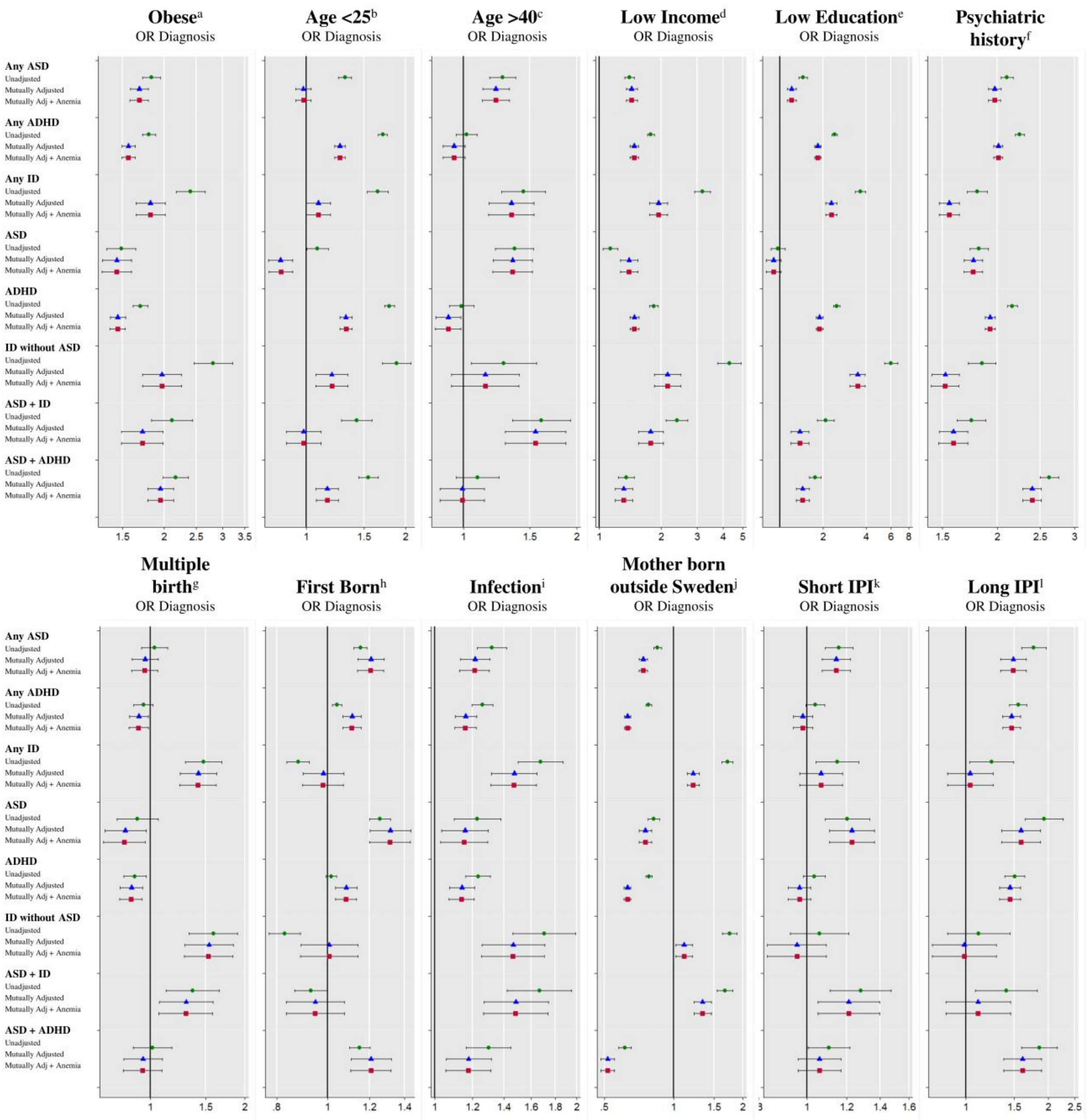

© 2019 Wiegersma AM et al. *JAMA Psychiatry.*

eFigure 1. ***The association between potentially confounding factors and risk of ASD, ADHD, or ID.*** The covariates were first examined in a generalized estimating equation (GEE) model to examine their relationship with the different outcome diagnoses in the GEE models with logit link clustered on maternal identification number (Unadjusted; green circles). This model was adjusted only for sex and birth year. A model including all covariates (Mutually Adjusted; blue triangles) was then used to understand the relationship between each covariate and each outcome after consideration of all other potential covariates. Finally, to examine a covariate´s association with the different outcome diagnoses after consideration of maternal anemia diagnosis and other covariates, maternal anemia diagnosis was added to these models (Mutually Adj + Anemia; red squares). (A) The risk of each outcome associated with being obese (compared to having a normal weight). (B) The risk of each outcome associated with having a mother younger than 25 years old at birth (compared to 30-34). (C) The risk of each outcome associated with having a mother of 40 years old or older at birth (compared to 30-34). (D) The risk of each outcome associated with a family income in the lowest quintile (compared to the highest quintile). (E) The risk of each outcome associated with the highest parental education level being less than ≤9 years of schooling (compared to >12 years of schooling). (F) The risk of each outcome associated with any maternal psychiatric history. (G) The risk of each outcome associated with a multiple birth. (H) The risk of each outcome associated with being first born (compared to being later born). (I) The risk of each outcome associated with mother hospitalized for infection during pregnancy. (J) The risk of each outcome associated with a mother being born outside of Sweden (compared to mothers born in Sweden). (K) The risk of each outcome associated with having a short interpregnancy interval (<1 year, compared to an IPI of 2-5 year). (L) The risk of each outcome associated with having a long interpregnancy interval (>10 year, compared to an IPI of 2-5 year).

© 2019 Wiegersma AM et al. *JAMA Psychiatry.*

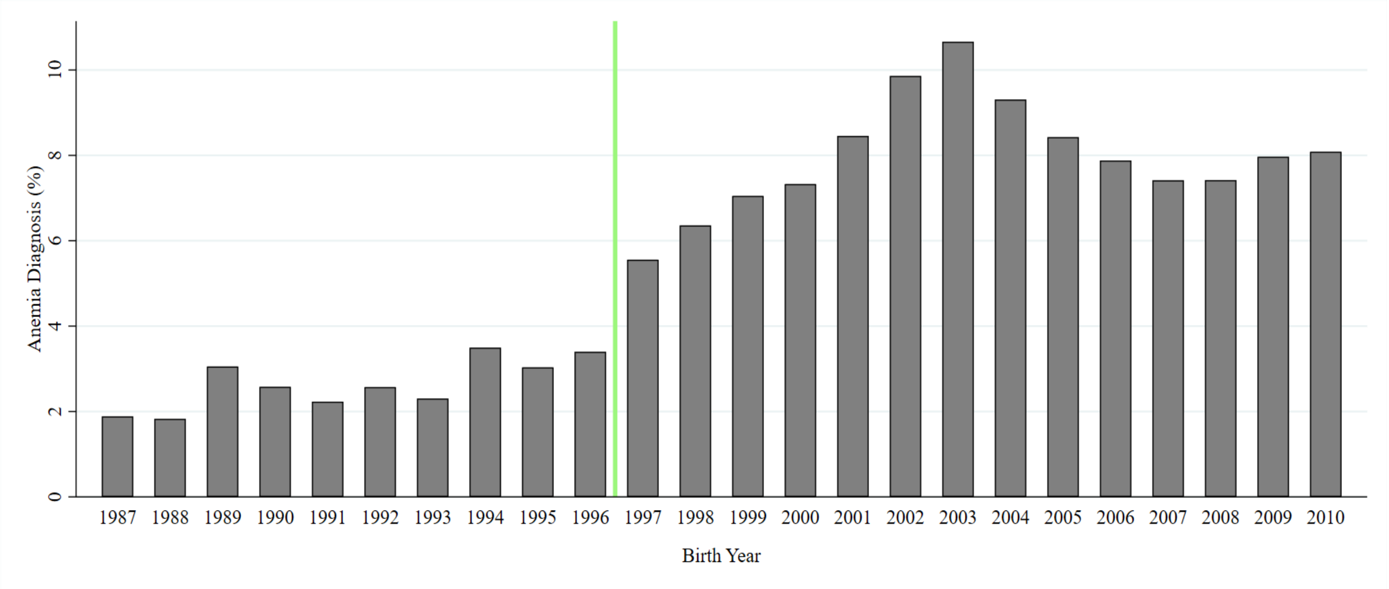

eFigure 2. ***Prevalence of maternal anemia per birth year***. A sensitivity analysis was performed stratifying the cohort on birth years before and after 1997.

© 2019 Wiegersma AM et al. *JAMA Psychiatry.*

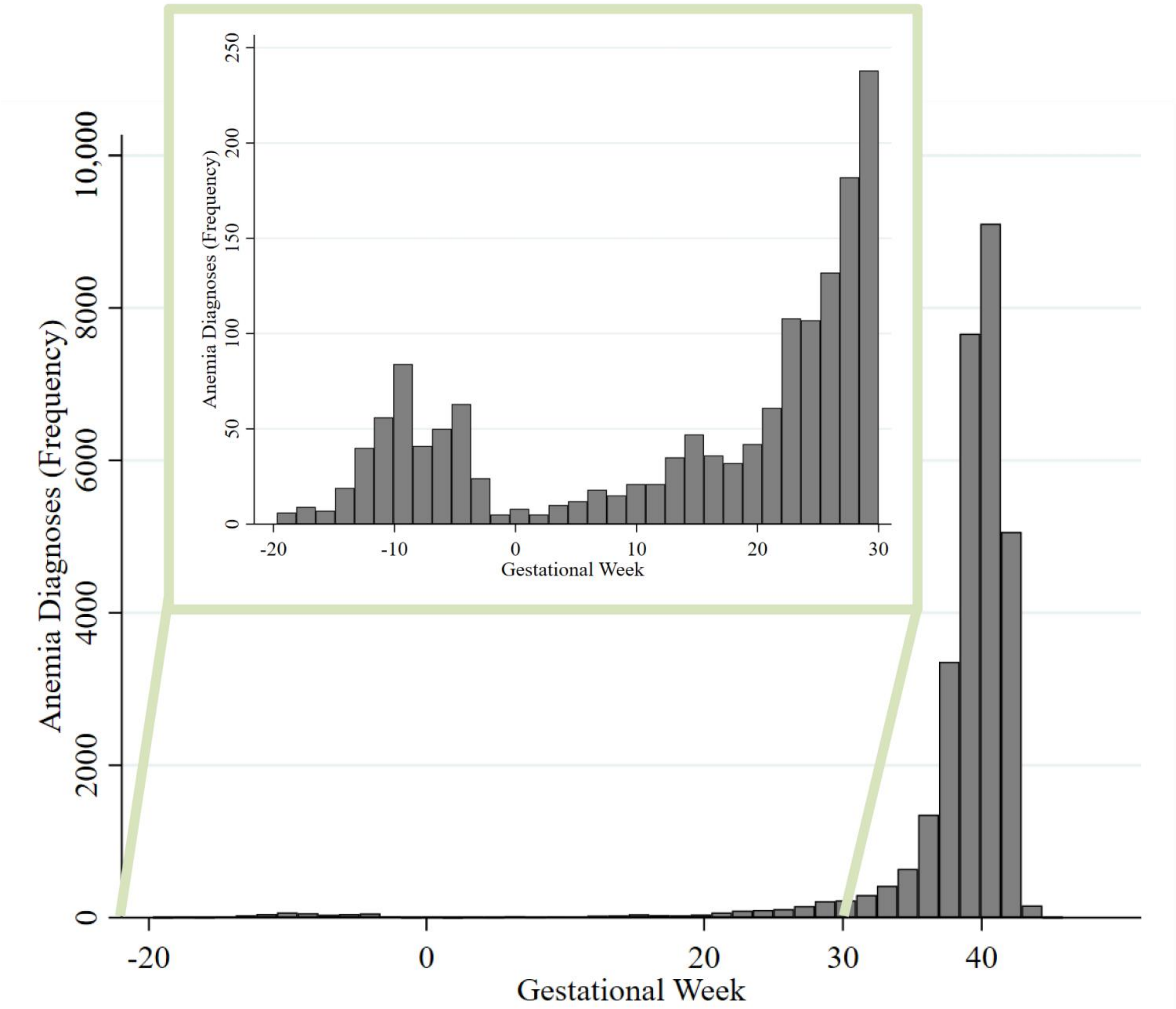

eFigure 3. ***Prevalence of maternal anemia per gestational week.*** Negative values on the x-axis represent weeks before gestation. The inset focuses on the range 20 weeks before gestation up until gestational week 30.

© 2019 Wiegersma AM et al. *JAMA Psychiatry.*

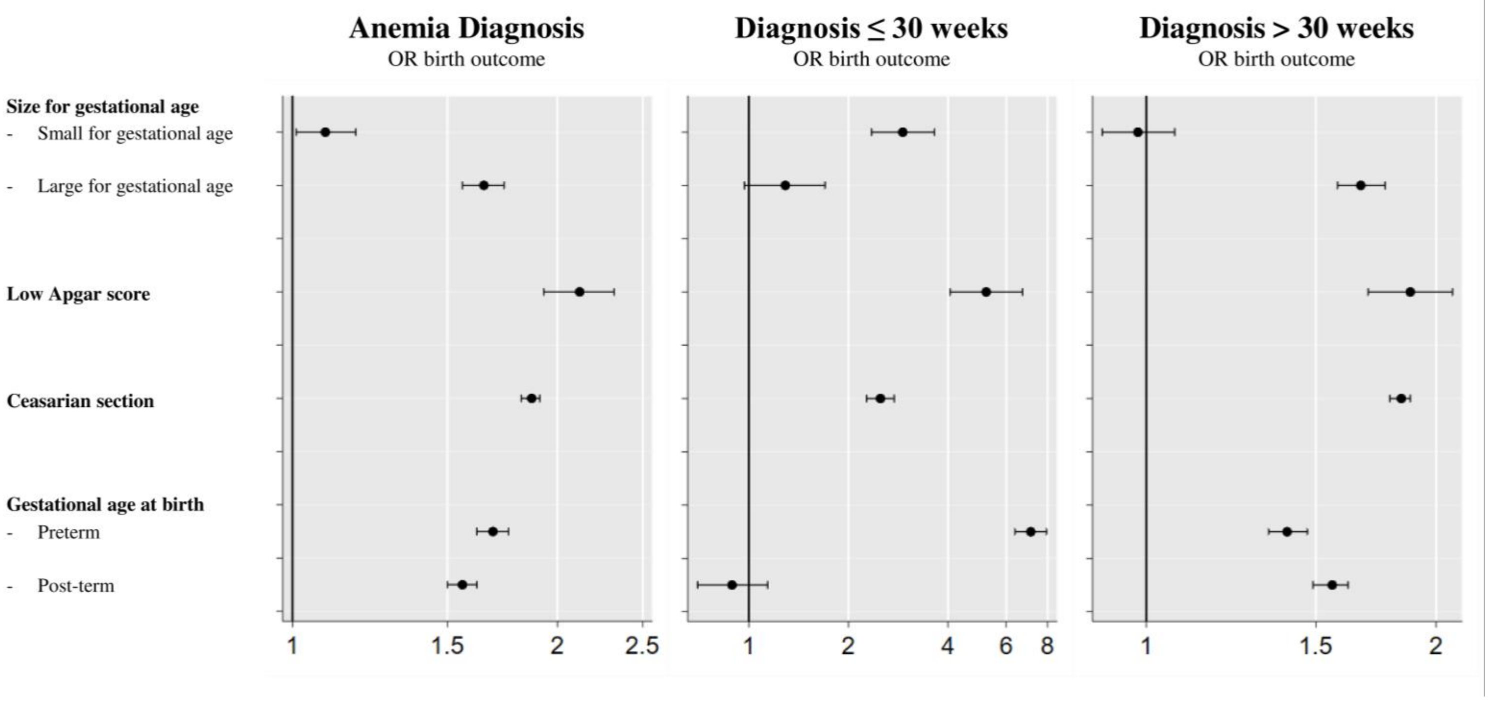

eFigure 4. ***Risk for pregnancy outcomes in relation to diagnosis of maternal anemia, comparing mothers diagnosed with anemia during pregnancy (at any point, diagnosed ≤30 weeks, or diagnosed >30 weeks) to mothers not diagnosed with anemia.*** Odds ratios and 95% confidence intervals were calculated via generalized estimating equation (GEE) modeling with logit link clustered on maternal identification number.

© 2019 Wiegersma AM et al. *JAMA Psychiatry.*

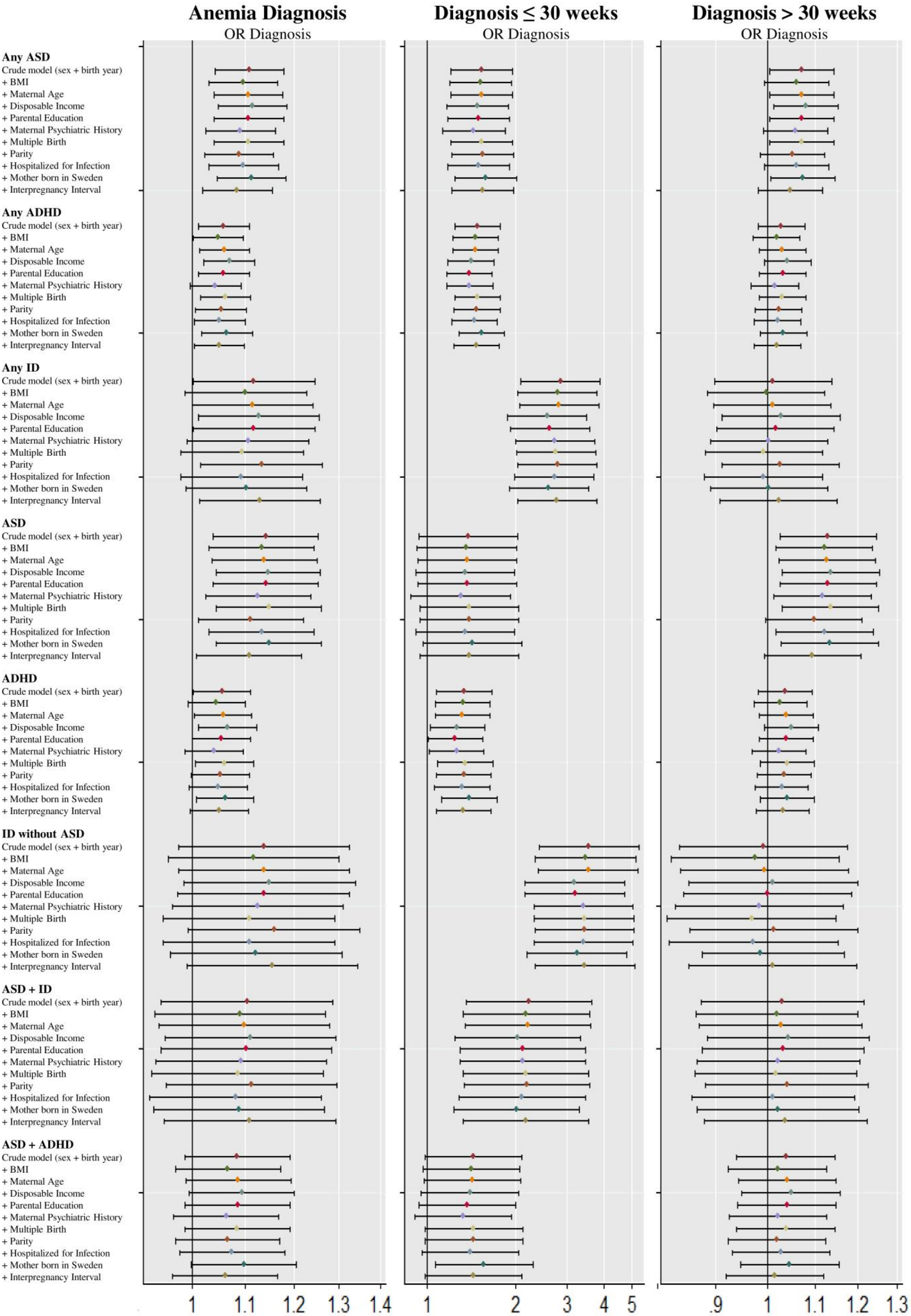

© 2019 Wiegersma AM et al. *JAMA Psychiatry.*

eFigure 5. ***An exploration of the influence of different potentially confounding factors on the risk for diagnostic outcomes related to any anemia diagnosis, anemia diagnosed ≤30 weeks and anemia diagnosed >30 weeks.*** In order to examine which covariate had the greatest modulating impact on the risk estimates, covariates were individually added to a crude model (already adjusted for sex and birth year). The odds ratios were calculated via generalized estimating equation (GEE) modeling with logit link clustered on maternal identification number. We tested the modulating effect of both parity and interpregnancy interval (IPI), but considered only IPI in the main model but considered only IPI in the main model because the two variables were related and co-linear.

© 2019 Wiegersma AM et al. *JAMA Psychiatry.*

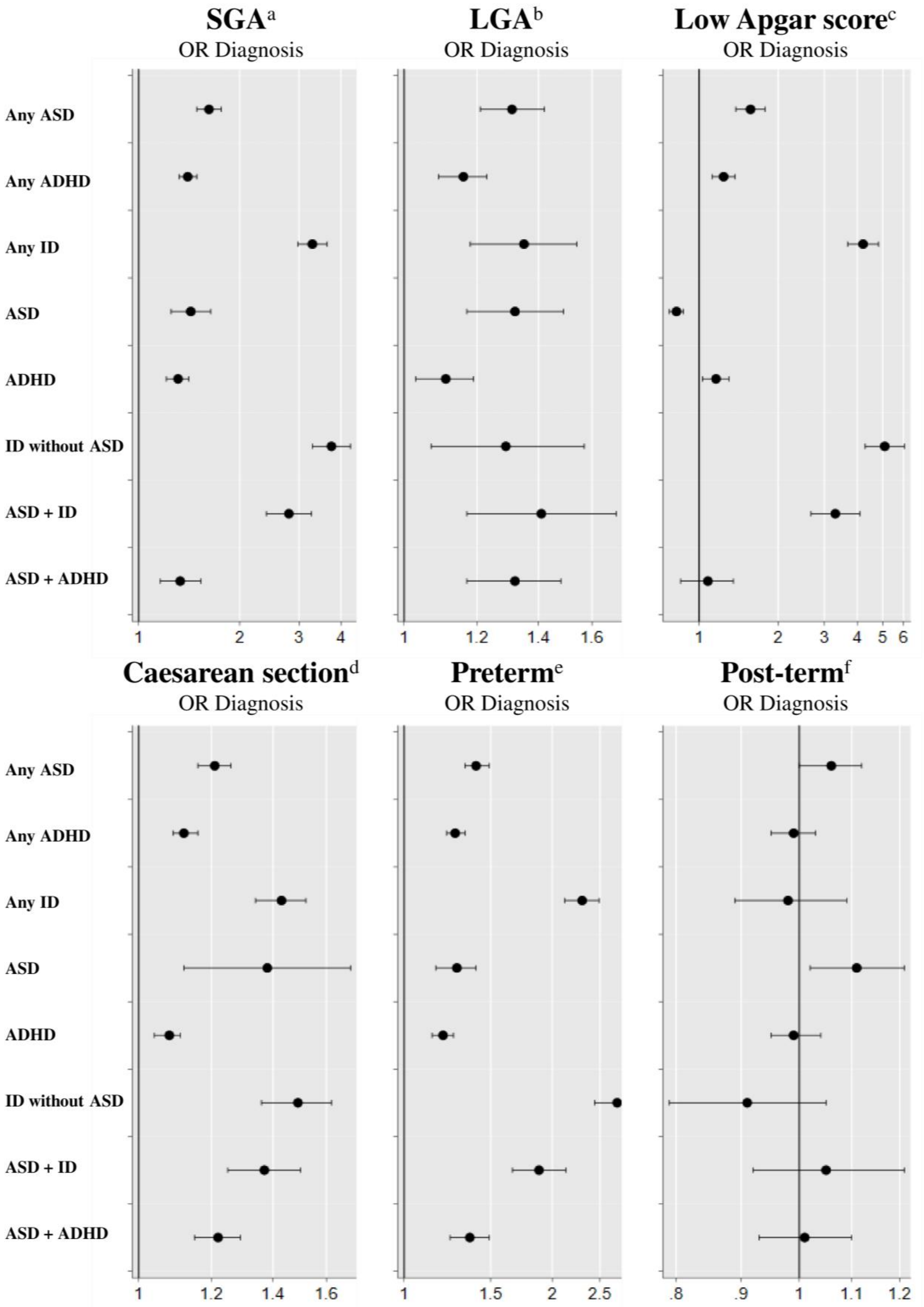

eFigure 6. ***Odds ratios (+95% confidence intervals) for neurodevelopmental disorders (ASD, ADHD and ID) in relation to potential mediators.*** Odds ratios were calculated via generalized estimating equation (GEE) modeling with logit link clustered on maternal identification number. The models accounted for sex and birth year. (A) The risk of each outcome associated with being born small for gestational age (compared to having a normal

© 2019 Wiegersma AM et al. *JAMA Psychiatry.*

size for gestational age). (B) The risk of each outcome associated with being born large for gestational age (compared to having a normal size for gestational age). (C) The risk of each outcome associated with having a low Apgar score (<7). (D) The risk of each outcome associated with being born via a caesarean section. (E) The risk of each outcome associated with being born preterm (compared to being born term). (F) The risk of each outcome associated with being born post-term (compared to being born term).

© 2019 Wiegersma AM et al. *JAMA Psychiatry.*
